# Photovoltaic fields largely outperform afforestation efficiency in global climate change mitigation strategies

**DOI:** 10.1093/pnasnexus/pgad352

**Published:** 2023-11-21

**Authors:** Rafael Stern, Jonathan D Muller, Eyal Rotenberg, Madi Amer, Lior Segev, Dan Yakir

**Affiliations:** Earth and Planetary Sciences Department, Weizmann Institute of Science, 7610001 Rehovot, Israel; Earth and Planetary Sciences Department, Weizmann Institute of Science, 7610001 Rehovot, Israel; Earth and Planetary Sciences Department, Weizmann Institute of Science, 7610001 Rehovot, Israel; Earth and Planetary Sciences Department, Weizmann Institute of Science, 7610001 Rehovot, Israel; Physics Core Facilities Department, Weizmann Institute of Science, 7610001 Rehovot, Israel; Earth and Planetary Sciences Department, Weizmann Institute of Science, 7610001 Rehovot, Israel

**Keywords:** climate change mitigation, afforestation, photovoltaic electricity, land-use change, radiative forcing

## Abstract

Suppression of carbon emissions through photovoltaic (PV) energy and carbon sequestration through afforestation provides complementary climate change mitigation (CCM) strategies. However, a quantification of the “break-even time” (BET) required to offset the warming impacts of the reduced surface reflectivity of incoming solar radiation (albedo effect) is needed, though seldom accounted for in CCM strategies. Here, we quantify the CCM potential of PV fields and afforestation, considering atmospheric carbon reductions, solar panel life cycle analysis (LCA), surface energy balance, and land area required across different climatic zones, with a focus on drylands, which offer the main remaining land area reserves for forestation aiming climate change mitigation (Rohatyn S, Yakir D, Rotenberg E, Carmel Y. Limited climate change mitigation potential through forestation of the vast dryland regions. 2022. Science 377:1436–1439). Results indicate a BET of PV fields of ∼2.5 years but >50× longer for dryland afforestation, even though the latter is more efficient at surface heat dissipation and local surface cooling. Furthermore, PV is ∼100× more efficient in atmospheric carbon mitigation. While the relative efficiency of afforestation compared with PV fields significantly increases in more mesic climates, PV field BET is still ∼20× faster than in afforestation, and land area required greatly exceeds availability for tree planting in a sufficient scale. Although this analysis focusing purely on the climatic radiative forcing perspective quantified an unambiguous advantage for the PV strategy over afforestation, both approaches must be combined and complementary, depending on climate zone, since forests provide crucial ecosystem, climate regulation, and even social services.

**Significance Statement**

Forests and photovoltaic (PV) fields both have significant potential for mitigating climate change, either through carbon uptake by photosynthesis or replacing emissions in energy production. However, both increase global heat load by darkening the surface, and a comparison of the time required to offset this warming impact is critical. Here, we quantify the climate change mitigation potential of both approaches, considering reduced atmospheric carbon, surface energy balance, and land area required to lower global carbon emissions across different climatic zones. Results show that in drylands, PV fields are >50× more efficient than afforestation. In more humid climates, afforestation advantage increases but demands more land than available. Nevertheless, forests provide many other ecological benefits compared with this pure climatic perspective.

## Introduction

Maintaining the average global temperature increase below the 1.5°C target which will maintain the Earth system boundaries at a stable state which will remain supporting human civilization (1, 2) demands a substantial and immediate reduction of carbon emissions in the energy sector (3, 4). Low-carbon photovoltaic (PV) electricity generation can significantly contribute to decrease anthropogenic carbon emissions (5, 6), but it requires large-scale infrastructures and is ultimately limited in capacity. Forest restoration (through afforestation of currently unforested land and reforestation of depleted forests) can play an important complementary role in climate change mitigation (CCM) efforts by providing an immediate mean for actively removing atmospheric carbon, which is considered an effective form of long-term carbon storage (3, 7–9). However, both large-scale PV electricity generation and afforestation projects have a high land area demand, which may lead to competition for land in areas that can support forests.

Drylands represent a large proportion of the Earth’s land surface, of which nearly half is semiarid (about 18% of the global land surface area, or about 30 Mkm^2^) and has attracted attention for potential afforestation projects, due to the availability of large nonforested areas (7, 10–13). However, they also have the potential to host a significant proportion of utility-scale PV fields, taking advantage of the low cloud cover, high insolation at low latitudes, low annual variation in day length, and much lower land-use competition with agriculture, cities, and industries (12–15). The alternative of local PV generation to supply each country's energy demand (rather than concentrating it in drylands) would require hundreds of thousands of square kilometers, greater than their entire urban areas and croplands in some cases, which is unfeasible in many countries (15, 16).

Covering typically bright and reflective drylands with dark PV panels (albedo, *α*: ∼0.05) (17), or dark forests (*α*: ∼0.10) (18, 19), can significantly increase surface temperature (*T*_s_) and, in turn, convective heating of the atmospheric boundary layer (20–24). This could counteract the benefits of reduced carbon emissions through PV electricity production (25) or carbon absorption through biomass accumulation in forests (9). Such albedo changes dramatically affect the surface energy budget (Eq. 1):


$$R_{n}=S_{\mathrm{in}}(1-\alpha )+L_{\mathrm{in}}-L_{\mathrm{out}}=H+\mathrm{LE}+G+PV_{e}+B,$$ (1)

where the net radiation at the surface (*R*_n_) represents the balance of the short-wave incoming solar radiation (*S*_in_) and the outgoing reflected part due to surface albedo, *α*, and the incoming thermal radiation emitted and reflected by the atmosphere to the surface (*L*_in_) and that emitted and reflected by the surface (*L*_out_). In the case of PV fields, some of the *S*_in_ is removed through conversion of energy to electricity, requiring an additional term, PV_e_ (equivalent to *S*_in_ × PV_eff_, i.e. the PV field's land efficiency; see Materials and methods). The surface energy load, represented by *R*_n_, is, in turn, dissipated by nonradiative fluxes, including sensible heat, *H*, latent heat, LE, some energy storage *G* on short time scales, and a small fraction used for biochemistry, *B* (Eq. 1).

In the low LE conditions of water-scarce drylands, the large heat load, enhanced by the albedo changes associated with afforestation or PV fields, contributes to atmospheric warming mainly via heat dissipation through *H*, which depends on the surface-to-air temperature difference (Δ*T*_s-a_) and the aerodynamic resistance to heat transfer (*r_H_*) (20, 21, 26, 27). Both afforestation and PV field installations can modify the surface *r_H_* in different ways that can be estimated based on measurements of *H* and Δ*T*_s-a_ according to Eq. 2:


$$r_{H}=\rho c_{p}\Delta T_{s-a}/H,$$ (2)

where *ρ* is the density (1.06 ± 0.03 kg m^−3^) and *c*_p_ is the heat capacity of air (1016 ± 3 J kg^−1^ K^−1^).

Notably, albedo-driven positive radiative forcing (RF_r_) directly impacts *R*_n_ and is a one-step change, having a global warming effect. In contrast, the negative RF achieved by a cumulative reduction of the carbon concentration in the atmosphere (RF_c_) due to carbon sequestration in afforestation is a cumulative term. By suppressing the future emissions of carbon from fossil fuel burning (carbon emission suppression [CES]), PV electricity generation results in a net reduction in atmospheric carbon when compared with the business-as-usual future scenario. The number of years, *n*, required for RF_c_ to balance RF_r_ can be estimated as *n* = RF_r_/RF_c_ and is termed the break-even time (BET; see Materials and methods).

Previous research on the effects of PV on climate using models and measurements (Table S1) inconclusively predicted an air temperature (*T*_a_) increase (23, 28, 29) or decrease (27, 30, 31). An *H* increase was effectively shown by measurements in Broadbent et al. (29), and surface temperature (*T*_s_) has been shown to decrease following PV deployment in deserts (32, 33).

Here, we aim to provide a comparative assessment of the BET of land-use change from open drylands to PV fields and afforestation, and an analysis of global climate zones, based on direct field measurements and a detailed analysis of the combined geophysical changes (in *α*, thermal radiation, *H*, and *r_H_*) and biogeochemical (carbon) climatic effects. This analysis also allows us to compare the land-based efficiency of PV and afforestation approaches as well as their effects on *T*_s_ and the potential effects on boundary layer dynamics. Combined, these comparisons can provide critical information for decision-making with respect to CCM by two of the major strategies available today.

## Results and discussion

### Surface energy fluxes

At our dryland site (see Materials and methods), installation of a PV field led to a ∼45% decrease in surface albedo (*α*: 0.17 ± 0.04 annual value integrated over the entire PV field; see Materials and methods) compared with the desert (*α*: 0.38 ± 0.02 annual value; see Materials and methods). The change in albedo (Δ*α*) was 0.16 ± 0.04 in the desert-to-forest transition and 0.21 ± 0.04 in the desert-to-PV field transition, in spite of the differences in absolute albedo values at the two sites (0.28 ± 0.03 and 0.38 ± 0.02 in the background annual values, respectively, due to an increased presence of shrubs and other soil properties outside the forest; Figure S1, Table 3).

**Fig. 1.**
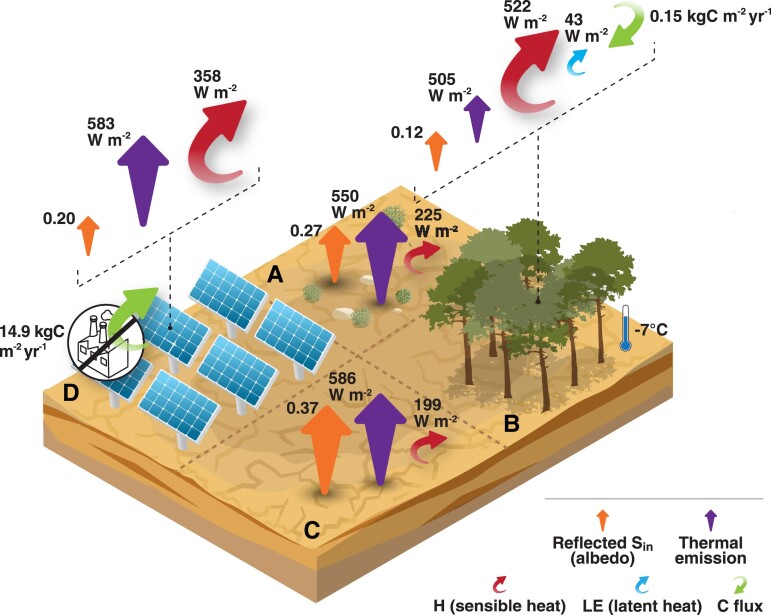
Land-use change effects. Desert-to-afforestation (A, B) and desert-to-PV (C, D) transitions in drylands, during summer midday. The size of the arrows represents the magnitude, with straight and round arrows for radiative and nonradiative fluxes, respectively. The green arrow represents carbon sequestration (photosynthesis) or suppression of emissions (represented by the thermoelectric power plant) in the afforestation and PV field, respectively. The thermometer represents the local cooling effect on surface temperature that the forest has.

Surface heating/cooling is revealed directly by *T*_s_, and indirectly by *L*_out_. We observed only a small (3 W m^−2^) and insignificant (*P* = 0.46) mean *L*_out_ difference between PV field and desert during summer midday hours (Table 1 and Figure 1). Accordingly, *T*_s_ was also very similar in the two sites (Table 1 and Figure 1) due to the similar *L*_out_ and emissivity (0.85 and 0.87 in the PV field and the desert, respectively; Methods S2). The difference in *L*_out_ between the PV field and desert was also not significant in the other seasons measured in this study: 11 W m^−2^ (*P* = 0.69) and 12 W m^−2^ (*P* = 0.9) in autumn and spring, respectively. In the Yatir afforestation, both *T*_s_ and *L*_out_ were significantly lower (*P* < 0.001) than in the surrounding desert by ∼7°C and 45 W m^−2^, respectively (Table 1). In spring, *L*_out_ difference between the afforestation and its desert background was 38 W m^−2^ (*P* < 0.01), and *T*_s_ difference was ∼5°C, although *P* = 0.96 (Table S4). No measurements were performed with the mobile station in the afforestation desert background in autumn. For the purpose of the RF_r_ calculation, an annual value of ∼10% of the Δ*L*_out_ of 18 W m^−2^ between the afforestation and the desert is considered to escape directly to space and participate in RF (34, 35) (Table 2; see Materials and methods). Compared with the practically unchanged *T*_s_ in the desert-to-PV field transition, this highlights the forest's efficiency in heat dissipation and its capability to decrease *T*_s_. In other words, the forest has a local surface cooling effect, while the PV field installation has no effect on *T*_s_ during midday hours.

**Table 1.** Geophysical effects of the deployment of PV field or forest in open drylands.

	PV		Afforestation	
Land use	PV field	Background	Forest	Background
*R*_n_ (W m^−2^)	529 (47)	410 (35)	650 (89)	452 (71)
*H* (W m^−2^)	358 (76)	199 (50)	522 (97)	225 (51)
LE (W m^−2^)	35 (28)	29 (25)	43 (48)	33 (42)
*L*_out_ (W m^−2^)	583 (16)	586 (26)	505 (15)	550 (17)
*T*_s_ (°C)	49.5 (2.2)	49.6 (3.7)	37.1 (2.5)	43.9 (2.5)
*T*_a_ (°C)	35.7 (2.6)	37.2 (3.0)	30.9 (2.3)	29.3 (1.9)
Δ*T*_s-a_ (°C)	13.8 (1.1)	12.4 (1.2)	6.3 (1.0)	14.5 (2.1)
*r_H_* (s m^−1^)	44.0 (12)	70 (15)	13 (3)	74 (25)

Values represent summer midday (10 Am–3 Pm) means, and SD is in parentheses. Air (*T*_a_, °C) and mean surface temperatures (*T*_s_, °C), surface–air temperature difference (Δ*T* = *T*_s_ − *T*_a_, °C), sensible heat flux (*H*, W m^−2^), and aerodynamic resistance to heat transfer (*r_H_*, s m^−1^) are shown for each land-use type.

**Table 2.** Geophysical and geochemical RF effects and the BET for three fossil fuel scenarios and the world mixture of fossil fuels.

	Coal	Oil	Gas	World mixture	Afforestation
CO_2_F (kgCO_2_ kWh^−1^)	0.87	0.67	0.40	0.54	N/A
CF (kgC kWh^−1^)	0.24	0.18	0.11	0.15	N/A
LCA (kgC kWh^−1^)	0.026				N/A
CF-LCA (kgC kWh^−1^)	0.21	0.15	0.08	0.12	N/A
CES or sink (kgC m^−2^ year^−1^)	26.0	19.3	10.2	14.9	0.15–0.28
RF_c_ (W m^−2^ × 10^−14^)	−7.15	−5.31	−2.81	−4.11	−4.12 × 10^−2^
RF_r_ local (W m^−2^)	65.66				39.38
RF_r_ global (W m^−2^ × 10^−13^)	1.01				0.76
Break-even time (years)	1.4	1.9	3.6	2.5	94–185

CO_2_F is the CO_2_ footprint (kg kWh^−1^), and CF is the carbon footprint (kg kWh^−1^), obtained from IRENA for fossil fuel scenarios for the year 2005 (the last year before renewables became larger than 1% of the global mixture) (6), and the conversion factor of C(kg) = CO_2_(kg) × 0.27. LCA of the PV panels is from da Silva Lima et al. (36) (see Materials and methods). CF-LCA is the carbon footprint of the PV panels after the carbon emitted during the panel's life cycle (mining, manufacturing, transport, installation, operation, and disposal or recycling and electricity storage in lithium-ion batteries) is deduced from its mitigation potential. CES is based on this PV field EP_a_ (EP_a_ = 123 kWh m^−2^ year^−1^). RF_c_ (W m^−2^) is the radiation forcing of the carbon component, while RF_r_ (W m^−2^) is the radiation forcing of the radiation components (albedo and longwave thermal radiation). CO_2_ and C footprints of fossil fuels and LCA of PV panels are not applicable to afforestation (N/A).

Neither *T*_a_ (5 m above ground level) nor Δ*T*_s-a_ above our PV field was significantly different from the adjacent desert (Table 1; Tables S3 and S4 and Discussion S1), in contrast to previous studies that showed a heat island effect (23, 26, 28, 29, 33) (Table S1). While this could be explained by some intrinsic differences between the sites (e.g. background vegetation removal reducing evaporative cooling and less dense PV installations (28, 33)), a possible underestimation of heat dissipating *H* fluxes in models is likely an important factor. Indeed, considering large *H* fluxes increased model precision considerably for the prediction of *T*_a_ and *T*_s_ related to PV systems in a previous study (37).

An *H* increase of 25–50 W m^−2^ was recently reported in a PV field in a desert in Arizona, United States (29). Our results show a more pronounced effect with an *H* increase of ∼160 W m^−2^ during summer midday hours in the desert-to-PV transition (Table 1). One of the main differences is that our study focused in the summer season, while Broadbent et al. (29) focused in the spring and under different insolation patterns. In afforestation, the mechanism behind the *H* increase of 297 W m^−2^ in the summer (Table 1 and Figure 1), despite the cooler surface, is associated with increased roughness and turbulence that decreases aerodynamic resistance to heat transfer, *r_H_* (20, 21, 26, 29), and has previously been termed a “convector effect” (22, 38). Note that the apparent contradiction—of the afforestation presenting half the Δ*T*_s-a_ of the PV field but ∼1.45× the PV *H* flux—is explained by an almost 3.4× lower *r_H_* (Table 1, Discussion S2).

Different effects of land-use change on *T*_s_, *H*, and on boundary layer depth (20, 21) can have important implications when considered at a large scale due to their potential impact on local and regional climate, wind convergence, and convection, with potential effects on cloudiness, rain, and temperature (11, 20, 23, 39). The *T*_s_ reduction achieved in the afforestation (Table 1 and Figure 1; Tables S3 and S4) should be considered critically, as the heat dissipation to the planetary boundary layer would still yield increased global RF, unless a short BET is achieved.

### BET

Based on the calculated CES and the PV life cycle analysis (LCA), we estimated the RF_c_ and, in turn, the BET for the PV installation in drylands, resulting in a range of 1.4–3.6 years for the three different fossil fuel scenarios (Table 2). In comparison, many hydropower reservoirs have a BET of 4–80 years while having an albedo lower than that of PV fields (0.08) (40). In contrast, the annual carbon assimilation rate of dryland forests—∼0.15 kgC m^−2^ year^−1^ for Yatir (41) or 0.28 kgC m^−2^ year^−1^ for global dryland forest generalization (7)—results in a BET which is up to 2× longer than the expected main forest C accumulation period (e.g. 80 years in Braakhekke et al. (42), Table 2).

A further comparison between PV fields and afforestation reveals stark differences in the land area required to offset carbon emissions from electricity production. The global electricity demand estimated for 2050 (6) is 55,000 TWh year^−1^, which would result in an emission of ∼6.6 GtC year^−1^, considering the global fossil fuel mix (Table 2; see Materials and methods). Sequestering this emission by dryland afforestation would require ∼26–44 Mkm^2^ (based on Qubaja et al. (41), or the estimated mean for global drylands (7), respectively), significantly more than the estimated 4 Mkm^2^ available for dryland afforestation (43). Emission suppression using PV would require a mere 0.44 Mkm^2^ in the same climate zone (0.75% of global drylands (12), Table 3), without considering the extra space needed to deal with production intermittency (15). This is mainly due to a 100× factor of the CES by PV energy versus annual photosynthesis of the nearby afforestation considered in this study (14.9 versus 0.15 kgC m^−2^ year^−1^, Tables 2 and 3 and Figure 1), with little difference in the albedo change. While any carbon uptake is considered important, we estimated here a long BET that could limit the land-based efficiency of afforestation as a useful CCM approach compared with PV fields in drylands. Therefore, we emphasize the complementary combination of reducing emissions through PV usage in drylands and further removal of atmospheric C into storage in biomass in the temperate and tropical zones could be a useful combination of CCM strategies. However, this assessment, based on the present study, could be sensitive to variations in climatic conditions (present and future ones), including teleconnection effects and ecohydrological feedbacks on both atmosphere and soil conditions.

**Table 3.** Global perspective of biogeochemical and biogeophysical effects of afforestation and PV field installation.

	Temperate	Tropical	Drylands
Afforestation			
*α*_forest_	0.14	0.13	0.12
*α*_background_	0.20	0.18	0.28
Δ*α*_background-forest_	0.06	0.05	0.16
C sequestration (kgC m^−2^ year^−1^)	0.31	0.41	0.15–0.28
Break-even time (years)	18.5	15.4	94–175
Available land area (Mkm^2^)	2.1	4.7	4
Required land area (Mkm^2^)	21.3	16.1	26–44
PV			
*α*_PV field_	0.13	0.10	0.17
*α*_background_	0.20	0.18	0.38
Δ*α*_background-PV_	0.07	0.08	0.21
CES (kgC m^−2^ year^−1^)	12.1	15.8	14.9
Break-even time (years)	<1	<1	1.4–3.6
Required area (Mkm^2^)	0.55	0.42	0.44
Area (m^2^)	110,000	130,000	75,600
*E*_g_ (kWh m^−2^ year^−1^)	900	1,500	2,122
EP (GWh year^−1^)	10	17	9.3
Land fraction of panels (*f*_pv_)	0.45	0.60	0.43
Panel efficiency (PV_eff,panels_)	0.15	0.14	0.12
Land efficiency (PV_eff_)	0.11	0.09	0.06
EP_a_ (kWh m^−2^ year^−1^)	90	130	123

Sites in the temperate (Germany) and tropical (Panama, Methods S3) zones were selected that had both forest and grassland ecosystems. Carbon sequestration by afforestation of temperate and tropical forests was obtained from Luyssaert et al. (44) and for dryland forests from Qubaja et al. (41) and Veldman et al. (9). Albedo (*α*) was obtained from the data available in Fluxnet and Euroflux networks and from the literature in the case of the tropical *α* for the forest and grassland (45). The reported values are medians, thus reducing the impact of extreme years. Percentages of PV panels and total areas were estimated from Google Earth (Methods S3). The estimated electricity generation by the PV field (kWh year^−1^) was obtained from the GPPD v. 1.3 database. EP_a_ (kWh m^−2^ year^−1^) was calculated using Eq. M5. Available land for afforestation was obtained from Griscom et al. (8) for temperate and tropical climates and from Rohatyn et al. (43) for drylands. The land areas required to offset 6.6 GtC year^−1^ are reported for all cases. Land areas necessary for afforestation are sensitive to carbon sequestration, here obtained from Fluxnet sites, but could nearly double using estimates for the entire climate zone by Griscom et al. (8).

Temperate and tropical regions have higher precipitation rates and are therefore more suitable for forest growth than drylands. Afforestation in these zones exhibits a significantly lower BET (15.4–18.5 years, Table 3) than in drylands and, in turn, a smaller land area required to sequester the carbon emissions (16.1–21.3 Mkm^2^, Table 3). However, this still vastly exceeds the available land area for afforestation in each climate zone, and complete afforestation of the available land in both regions would achieve less than half of the carbon sequestration required (2.6 GtC year^−1^) (8) to balance anthropogenic fossil fuel emissions. At a global scale, the entire area available for forest restoration would have to be used (18 Mkm^2^ of forest cover) (7), representing ∼40× more land area than that required by PV in drylands.

PV fields are less sensitive to their location, i.e. neither the BET nor the land area required for CES differs significantly among climate zones (Table 3). The tropical and temperate zones have higher cloud cover and lower solar angles with shorter insolation hours during a significant part of the year. Nevertheless, the resulting reduction in insolation was likely outweighed by the angle and density of PV installation and/or different technologies that increase panel efficiency (Methods S3). Therefore, despite lower annual electricity production (EP_a_) in the temperate zone, the gross land efficiency for electricity generation of PV fields was highest there (Table 3). Although the BET of PV fields in these regions is very small due to a much lower Δ*α* effect (<1 year, Table 3), installation could lead to further carbon emissions related to impacts on background vegetation (15).

Indeed, PV electricity generation can prevent much higher carbon emissions than the amount that afforestation can sequester. However, the active removal of past emitted carbon and the possible long-term storage as biomass and soil organic matter provides an important advantage for afforestation. Moreover, it is important to consider that our study presents a purely climatic perspective, as forests can provide a variety of additional natural and cultural ecosystem services that are not provided by industrial PV systems (e.g. biodiversity, green space, soil protection, wood production, recreation, tourism, mental and physical health, and other societal needs) (46–48). Hence, these should be considered in policy and management decisions in a broader perspective of CCM plans.

Besides carbon emission, other environmental impacts associated with the installation of PV fields in large ecosystem areas can occur to a different degree, such as soil erosion, dust production, increased risk of fires, water pollution and soil contamination, noise and light pollution, alteration of nutrient dynamics, biodiversity loss, heavy metals released to the environment (48, 49), and even sociological and cultural impacts (46). Nevertheless, since the land required for PV is much smaller than that needed for afforestation in drylands for CCM, the scale of the impacts in these areas would be significantly lower. Note also that some forms of dryland afforestation also have significant biodiversity impacts (50, 51).

In conclusion, we note that the large dryland fraction of Earth's land surface must be considered in developing any large-scale CCM strategy. In this context, drylands offer the potential for both PV and afforestation, and we offer a much-needed comparative, quantitative analysis of both strategies. We show that PV clearly provides greater land-use efficiency in drylands, but some of the advantages of afforestation (e.g. long-term C storage or surface cooling) should not be neglected.

## Materials and methods

### Measurement location

The research part concerning the PV fields’ impact on drylands was conducted in a PV field in a hyperarid region in the Arava valley in Israel, next to kibbutz Ketura (Figure S1). The mean annual rain is about 28 mm, with an average of 1.3 days of rain events above 5 mm, aridity index < 0.03. The mean annual temperature is 23.9 ± 8°C for the years 2001–2019, with mean daily maximum summer temperatures (July and August) reaching 38.9 ± 1.8°C and a mean daily winter minimum of 9.5 ± 2.8°C (January and February) (Israel Meteorology Service). The annual average of the incoming shortwave radiation is 245 W m^−2^ (Israel Meteorological Service) or 2,122 kWh m^−2^ (52).

The PV field chosen has an area of 75,600 m^2^ and an installed production capacity of 4.9 MW. Rows of monofacial nontracking crystalline silicon PV panels intercalated with soil compose 84% of the field (from which 51% are PV panels and 49% is soil, Figure S3), while 16% of the field area is composed of infrastructure (roads and buildings, measured from satellite images in QGIS software). Considering the whole PV field with the PV panels and soil rows, and the necessary infrastructure, the total coverage of the panels is 43% versus 57% of mainly bare soil. This PV field is about 400 m long and has a maximum width of 250 m. Each panel has an extension of 5 m vertically, 2 m horizontally, and 0.05 m width. All PV panel rows are fixed and facing south. The height between the top of the panel and the ground is 3 m, and the bottom is 0.5 m above ground. The gap between each of the lines of panels is 5 m, and the inclination angle is 30°.

The afforestation data were measured in a fixed eddy covariance (EC) station at the Yatir forest (41) (Figure S1). It is located in a 2,800 ha afforestation of mainly *Pinus halepensis* trees with a height of ∼10 m and a 50% canopy cover (estimated from Lidar data, June 2019) in the dry southern Mediterranean region, at the northern edge of the Negev desert in Israel (31°20′49″N; 35°03′07″E; altitude 600 to 850 m above sea level; cf. Methods S6) (41, 53). The site contains an EC flux tower operating since 2001, with EC sensors ∼10 m above canopy. Up- and downwelling shortwave (0–4,000 nm, W m^−2^; CM21, Kipp & Zonen B.V., Delft, The Netherlands) and longwave radiation (4–50 µm, W m^−2^; Eppley, Newport, RI) were measured at the same tower, ∼5 m above canopy.

Note that the drylands considered in this study (from the semiarid to arid transition zone suitable for forestation or PV and the hyperarid suitable mainly for PV) represent the vast majority of global drylands from the climatological perspective (Figure S1f) and the global range of dryland surface albedo (Methods S1) (54).

### Mobile system measurements and data analysis

The mobile system was previously used and described in a number of recent studies (21, 35, 55–61). It consists of a full EC (62) and meteorological station transported by a 12-ton, 4WD truck (Figure S4), which provides air-conditioned instrument facility, internet communication, and a uninterrupted power supply (UPS) system that ensures the energy stability. It was powered by the electricity of a nearby kibbutz (Ketura) during the PV desert background measurements, through an infrastructure building of the Arava Power Company inside the PV field, as well as a generator during measurements in deserts outside the Yatir forest. Sensors include a sonic anemometer (R3-100, Gill Instruments, Lymington, Hampshire, United Kingdom) for 3D wind speed components (horizontal *u* and *v*, and the vertical *w*) and temperature derived from the speed of sound (63), an enclosed-path CO_2_/H_2_O Infrared Gas Analyzer (IRGA; LI-7200, LI-COR Lincoln, NE, United States), air temperature, relative humidity (HMP45C probes, Campbell Scientific, Logan, UT, United States), and pressure sensors (Campbell Scientific, Logan, UT, United States). Radiation sensors included the shortwave solar (0.28–2.8 µm, factory calibrated to the full 0 to 4 µm range; CMP21, Kipp&Zonen, Delft, The Netherlands) and longwave or thermal radiation (4.5–42 µm factory calibrated to the full 4–50 µm range; CGR-4, Kipp&Zonen Delft, The Netherlands). All the sensors were placed on top of a pneumatic mast that connects to the truck, which was positioned up to 35 m away from the truck in the center of the field sites with location and height aimed to provide a sufficient “fetch” for the EC system (Figure S5). The mast height was 2.7 m (spring), 4.5 m (autumn), and 5.1 m (summer) in the PV field desert background, 5.2 m (spring), 4.2 m (autumn), and 4.5 m (summer) in the PV field, and 5.0 m (2013) and 4.6 m (2015) at the Yatir desert sites. The measuring height of the sonic anemometer is located 0.63 m above the mast height. Raw EC data were collected using a custom LabView software (developed by Enrico Segre, 2010), and meteorological data were collected by a Campbell Scientific CR3000 logger (Logan, UT, United States). The IRGA was cleaned and calibrated for zero and span values before each season. A metal box deployed between the truck and mast hosts the IRGA's air pump (10 lpm flow) and the data logger for the radiation and the temperature and humidity sensors. More details about the mobile laboratory, with validations and calibrations, can be found in Asaf et al. (55) and Helman et al. (60). Soil heat storage (*G*) was not measured in this study.

Field measurements were performed in spring (2018 March 18–21 in the desert background and 2018 March 22–27 in the PV field), autumn (2018 October 16–23 in the desert background and 2018 October 24–31 in the PV field), and summer (2019 July 09–15 in the desert background and 2019 July 16–24 in the PV field), therefore covering different meteorological conditions and solar radiation incidence and sun angles. The reported dates correspond to full days with all the half-hour averages included (Table S2). Campaign-based measurements were proven to be a valid methodology in order to provide seasonal comparison between different land uses (21, 35, 55–61). The data for each campaign were continuous, with <3% of half-hourly gaps filled using the Marginal Distribution Sampling tool in Tovi. Comparisons with nearby long-term meteorological stations show that *S*_in_ and *T*_a_ conditions were well representative of each season (Figure S2).

Sensible (*H*) and latent heat (LE) flux data collection, processing, and postprocessing were performed according to established methodology (64–66). It included quality control flagging (67), sonic anemometer double rotation tilt corrections (68), and compensation of air density fluctuations due to water vapor variations (69–71), as well as spectral corrections of EC data (72, 73) and footprint and shear velocity (74, 75) estimation (Figure S6). We used the EddyPro 7.0.5 (LICOR Biosciences, Lincoln, Nebraska) and the novel Tovi 2.8.1 software (LICOR Biosciences, Lincoln, Nebraska). No air heat storage correction was applied since it was assumed to be negligible in magnitude. Due to the very open “canopy” characteristic of our measurement sites with the mobile station (PV field and desert backgrounds of the PV field and afforestation), total flux errors derived from storage are expected to be small (76).

EC flux measurements can be susceptible to bias due to instrument location, which we rigorously tested: Firstly, stable structures can result from the module arrangement, which can enhance convection (77). However, an analysis of cospectra provides confidence in our measurements and does not indicate directional effects. Secondly, an estimation of the random error in flux measurements (78) showed a mean error of 10 W m^−2^ in the desert background and in the PV field for *H* and 7 W m^−2^ for LE. Such uncertainties have previously been described for only the highest quality data (67), which adds confidence to our measurements. Such uncertainties can influence the precision of the energy balance estimations but not the conclusions of a clear *H* enhancement over the PV field. Additional future studies are required to better understand the effect of geometrical arrangements on fluxes.

The effect of shortwave radiation on the longwave radiation sensors was estimated using Eq. M1, obtained by a shading experiment as described in Pérez et al. (79):


$$\Delta L=0.38+0.01S_{in},$$ ( M1)

where *S*_in_ is the shortwave radiation measured by the mobile system radiation sensor, and the corrected longwave radiation value is obtained by subtracting Δ*L* from the raw longwave radiation sensor measurement. This equation is applied both for the incoming (downwelling, *L*_in_) and for the outgoing (upwelling, *L*_out_) radiation components.

Mean seasonal and annual albedo values were calculated as the ratio between incoming (downwelling, *S*_in_) and outgoing (upwelling, *S*_out_) shortwave radiation $\left(\alpha =\;\frac{S_{\mathrm{out}}}{S_{\mathrm{in}}}\right)$, on a half-hourly basis. PV desert background albedo showed negligible variations throughout the year due to the complete absence of vegetation (spring: 0.40; summer: 0.37; and autumn: 0.38). Annual albedo for the desert background (for RF calculation, see below) was calculated as the mean of all data, containing the combined data sets of all seasons. PV field albedo could not be measured directly due to height restrictions imposed by the field operator that were sufficient for flux data but not for radiative data (Methods S4). For this reason, PV field albedo was obtained through a combination of albedo data of its components and their fraction, as viewed from nadir: We used a set of drone imagery taken over the PV field throughout the day during the summer and autumn campaigns to measure the fraction of PV panels as well as exposed and shaded soil. Then, a cosine curve was fitted to each campaign's shade fraction as a function of time, with an additional set of cosine curves fitted to the yearly course as a function of day of year, in order to obtain the shaded soil fraction for each half-hour of the year. The PV panel fraction was a fixed 51%, and the remaining fraction was exposed soil. A value of 0.05 for PV panel *α* (17) was combined with the albedo of each time of the day of the desert background *α* for exposed soil. For shaded soil, the exposed soil *α* was multiplied by the diffuse radiation fraction (Methods S5) obtained from above-canopy measurements at the nearby Yatir station. Finally, seasonal and annual *α* were calculated as weighted means by *S*_in_ across the seasonal campaigns or the entire year, respectively.

### RF and “BET” calculations

The balance between the negative RF due to CES of PV power generation or carbon sequestration by forests (RF_c_; cumulative over time) and the positive RF due to changes in the surface energy budget of PV fields and forests (RF_r_; a one-time change) is used to estimate the BET required to balance the two effects (Eq. M2).


$$nRF_{c}=RF_{r}(W\;m^{-2}),$$ ( M2)

where *n* is the number of years to reach a balance. RF_r_ is defined as follows:


$$RF_{r}=\;\frac{[\Delta \alpha \;E_{g}]+0.1\Delta L_{\mathrm{out}}}{A_{E}}\;\;(W\;m^{-2}),$$ ( M3)

where Δα is the albedo change of the desert-to-PV or desert-to-forest transition. When the local effect is considered, the efficiency of the land area of a PV field in generating electricity (PV_eff_) additionally needs to be subtracted from Δα to account for the amount of incoming solar radiation that leaves the field as electricity and therefore reduces the local *R*_n_ (net radiation at the surface) (Eq. 1). We note that the land efficiency of a PV field is different from the panel efficiency (Methods S6 and below). *E*_g_ is the global radiation of the region (245 W m^−2^), and Δ*L*_out_ is the change in upwelling longwave radiation of this land cover change multiplied by the fraction directly emitted to space (∼10% (34)). Δ*L*_out_ was not significant in the desert-to-PV transition (Results and Discussion S2) and was therefore only relevant in the RF_r_ estimation of afforestation (annual scale: 18 W m^−2^) (35). *A*_E_ is the surface area of planet Earth (5.1 × 10^14^ m^2^), needed in order to obtain the global effect and to compare with the effect of the RF of CO_2_ (RF_c_) (25). The estimation of RF_c_ is based on the update by Etminan et al. (80) of the original equation of Myhre et al. (81) as used in Betts et al. (82):


$$RF_{C}=[a_{1}{\left(C-C_{0}\right)}^{2}+b_{1}|C-C_{0}|+c_{1}\bar{N}+5.36]\ln\;\left(\frac{C}{C_{0}}\right)\;(W\;m^{-2})$$ ( M4)

where *a*_1_ = −2.4 × 10^−7^ W m^−2^ ppm^−1^, *b*_1_ = −7.2 × 10^−4^ W m^−2^ ppm^−1^, *c*_1_ = −2.1 × 10^−4^ W m^−2^ ppm^−1^, *C*_0_ is the background CO_2_ concentration of the atmosphere (409.85 ppm on average in 2019), *C* is the new CO_2_ mixing ratio after the forcing caused by the land-use change (Δ*C* + *C*_0_), and $\bar{N}$ is the background N_2_O mixing ratio of the atmosphere (331 ppb on average 2019). CO_2_ and N_2_O average background mixing ratios for the year of 2019 (the year we finished our measurements) were obtained from NOAA/GML (https://gml.noaa.gov/ccgg/trends/). Δ*C* was obtained from the expression Δ*C* = *E* × AF/*k*, where *E* is the emission suppression (CES) in the case of PV installations, or carbon sequestration for an afforestation, AF is the carbon airborne fraction (0.46) (3), and *k* is the conversion from kilograms of carbon (kgC) to parts per million (ppm) (2.16 × 10^12^) (25). Note that this AF means that with regard to the effects on atmospheric carbon, any addition or removal of *C* is translated by the AF factor of 0.46 in terms of the atmospheric *C* pool due to the *C* partitioning among the atmosphere, land, and oceans. In the case of PV fields, *E* must be estimated from its EP_a_ reduced by the carbon emitted during a panel's life cycle and energy storage (LCA, see below).

In order to estimate the EP_a_ of a PV field per square meter, we used the following:


$$EP_{a}=\frac{365\cdot EP_{d}}{A_{\mathrm{pv}}}\;=\frac{365\cdot 25,500}{75,600}=123\;\mathrm{kWh}\;m^{-2}\;\mathrm{yea}r^{-1},$$ ( M5)

where EP_d_ is the annual mean of the daily electricity production provided by the PV field operator (25,500 kWh day^−1^) and *A*_pv_ is the area of the PV field (75,600 m^2^), multiplied by 365 days per year to obtain an annual estimate. In the case of our PV field, EP_a_ was about 123 kWh m^−2^ year^−1^. Since this is the real production that the PV field operator gets as a final product, it already includes losses related to the performance ratio (PR)—which ranges typically between 0.85 and 0.75 (83–86)—and that comprises losses related to the DC–AC current inverter, cables, and transformers (83, 87). Therefore, EP_a_ can be used directly to estimate the efficiency of the PV field. The PR is mainly influenced by temperature (14), and values provided by the operator of our study site fluctuated between >0.85 in the spring and slightly below 0.80 in the summer. Note that the PV panels’ high temperature (almost 60°C during peak, Discussion S1) also affects efficiency, and there is a decrease of ∼0.5% of the efficiency for every degree increase above 25°C (88–90). Nevertheless, PV fields can operate at a much higher temperature than forests, as those are physiologically limited to a maximum of 40–50°C (91) and require water for carbon sequestration and therefore are negatively affected by decreasing precipitation. In contrast, PV fields benefit from increased insolation resulting from lower cloudiness. The annual CES was estimated by multiplying EP_a_ by the annual carbon emission per kilowatt hour produced according to each fossil fuel or mixture of fuels (Table 2).

The CO_2_ footprint (CO_2_F, which corresponds to the emission of CO_2_ for each kilowatt hour produced) for each fuel type was obtained from the IEA (92). The impact of replacing thermoelectric electricity generation with PV generation was analyzed for three different fossil fuel sources—coal, oil, and gas, or a global average mixture of fuels (mainly coal, oil, and natural gas). We used the global average mixture of fuels from the year 2005 because it was just before the beginning of the large-scale deployment of renewable energy sources, weakening the signal of fossil fuel burning replacement. The carbon footprint (CF) was obtained by multiplying the CO_2_F values by a factor of ∼0.27 (molar mass C/molar mass CO_2_). The CES of each scenario of fuel replacement by PV electricity production (coal, oil, or gas thermoelectric power plants) is obtained according to Eq. M6:


$$\mathrm{CES}=EP_{a}(\mathrm{CF}\text{-}\mathrm{LCA}),$$ ( M6)

where CES is expressed in units of kgC m^−2^ year^−1^, and LCA is the life cycle analysis of PV installations. CES included the following: (i) EP_a_ per surface area of the PV field as a whole, including rows of soil between panels and infrastructure (EP_a_ of ∼123 kWh m^−2^ year^−1^ in our study site). This is mostly determined by the efficiency of the entire PV field (PV_eff_ = 5.8%) and includes losses due to the PV PR (i.e. the DC–AC current inverter, losses due to cables and transformers, and temperature-related efficiency decreases (93)) and the field cover parcel of the PV panels (43% in our case). (ii) The CF (i.e. emission of C per kilowatt hour produced) of fossil fuel energy sources that are replaced by PV for three scenarios of electricity production, i.e. either replacing 100% coal or gas production or that of the average global energy mix (92), providing the perspective of replacing mainly fossil fuels before the recent increase in alternative energy use. (iii) The carbon emission of PV electricity generation, based on a LCA that includes emissions from mining, manufacturing, transport, installation, operation, and disposal or recycling of PV panels and storage methods (36) (here considering lithium-ion batteries as the most used form of storage due to its space efficiency and energy density) (36). LCA values for PV electricity are typically in the 0.010–0.050 kgCO_2_eq kWh^−1^ range (25, 83, 94–96) before considering storage. The 0.039 kgCO_2_eq kWh^−1^ value (94) has a high resemblance to the conditions at our study (PV efficiency = 6% and irradiance 1,935 kWh m^−2^ year^−1^). When storage is added, a 0.095 kgCO_2_ kWh^−1^ LCA value is obtained (36), corresponding to 0.026 kgC kWh^−1^. Note that energy storage includes losses of between 5 and 40% in different storage options (36), but due to the high uncertainty and the possibility of demand side management of electricity use, it was assumed to be a minor component and was not included. Therefore, the LCA value of 0.026 kgC kWh^−1^ was used in this study (36).

Based on the EP_a_ of the PV field of this study (123 kWh m^−2^ year^−1^), and on the annual solar irradiance of the region (2,122 kWh m^−2^), we estimated the mean annual land-use efficiency of this entire PV field (PV_eff_) at ∼5.8%$\left(=\frac{EP_{a}}{E_{g}}=\frac{123}{2,122}\right)$. Note that the efficiency of PV panels at this field (PV_eff, panel_) is comparable to other different locations due to the different panel inclination angles (Methods S3). This value represents the actual ability to convert the incoming radiation to electricity (97) and was the same even when calculated separately for each season. It considers the diverse losses after the electricity generation, including the PR. It is important to note that this efficiency is for the PV field as a landscape unit, with all its different components (PV panels, sun-exposed soil and shaded soil, and infrastructure), because the focus of this study is to compare it with an alternative land use such as afforestation for carbon mitigation purposes. All calculations were done in Python using the numpy (98) and gmpy2 (99) libraries for precision purposes.

Detailed thermal radiation data were obtained through a deep learning segmentation algorithm for the different elements of the PV field (Figure S7 and Methods S4): PV panels, sun-exposed soil, and shade, and combined with ecosystem emissivity (Methods S2). These measurements were compared with data of continuous carbon and energy fluxes at the permanent forest flux measurement site (IL-Yat, http://www.europe-fluxdata.eu/home/sites-list) in the nearby semiarid Yatir pine forest plantation (Figure S1) (41). Desert background data both south (August 2013, March 2014) and north (August 2015, March 2016) of the forest were also obtained using the mobile system. We combined these analyses with two additional climate zones (temperate and tropical) using data obtained from other flux stations, analyses of satellite images, and data gathered from the literature (Methods S3).

## Supplementary Material

pgad352_Supplementary_Data

## Data Availability

The data underlying this analysis are freely available from the following sources: flux data for the sites in Israel: https://doi.org/10.34933/403d99aa-17e2-4f67-95a4-5de13681439c; GPPD database (v.1.1.0): http://datasets.wri.org/dataset/globalpowerplantdatabase; Fluxnet (https://fluxnet.org/); and Euroflux (http://www.europe-fluxdata.eu/). The Python scripts used to analyze the data are freely available under https://github.com/rafastern/Dryland_PV_vs_afforestation.
