# Supplementary material for: Photovoltaic fields largely outperform afforestation efficiency in global climate change mitigation strategies
Source: PNAS Nexus. 2023 Nov 21;2(11):pgad352. doi: 10.1093/pnasnexus/pgad352 (PMC10662455; doi:10.1093/pnasnexus/pgad352)
Supplement: pgad352_Supplementary_Data [file pgad352_supplementary_data.pdf]

Supplementary Information

Photovoltaic fields largely outperform afforestation efficiency in global climate-change mitigation strategies

Rafael Stern[1‡], Jonathan D. Muller[1‡], Eyal Rotenberg[1*], Madi Amer[1], Lior Segev[2], and Dan Yakir[1]

[1] Earth and Planetary Sciences Department, Weizmann Institute of Science; Rehovot, 7610001, Israel.

[2] Physics Core Facilities Department, Weizmann Institute of Science; Rehovot, 7610001, Israel.

‡ These authors contributed equally to this work

*Corresponding author: Eyal Rotenberg (eyal.rotenberg@weizmann.ac.il)

This file includes:

Supplementary Methods

Supplementary Discussion

Supplementary Figures

Supplementary Tables

Other Supplementary Materials for this manuscript include the following:

Data and code available on Github:
https://www.github.com/rafastern/Dryland_PV_vs_afforestation and
https://doi.org/10.34933/403d99aa-17e2-4f67-95a4-5de13681439c

Supplementary Methods

Methods S1: Site albedo in context of the global range of drylands albedo

Global dryland albedo values were obtained from a weighted global dryland albedo average calculated based on data published by Tsvetsinskaya et al., 2006 (Tsvetsinskaya *et al.*, 2006), which contains a set of tables with albedo values for each continent and soil type, along with the number of satellite imagery pixels that represent the area of each. A weighted average of global dryland albedo for the shortwave range (300-5000nm) by area was calculated based on this data along with standard deviation (0.32±0.07), maximum (0.43) and minimum (0.20).

The desert background albedo measurement from our campaigns were 0.38±0.02 at the PV site and 0.28±0.03 at the forest site, whereby the two sites are both within this global range, and the global mean is approximately half-way between the two sites.

Methods S2: Ecosystem emissivity

Obtaining land surface temperatures (LST) from remotely sensed upwelling longwave radiation (L_{out}) at Eddy Covariance towers requires accurate knowledge of plot-scale emissivity (ε) and downwelling longwave radiation (L_{in}) However, plot- to ecosystem-scale emissivity depends not only on the emissivity of the materials making up the ecosystem itself (e.g., soil, rocks, leaves), but also ecosystem structure, as that affects the emission of longwave radiation. Therefore, measurements of these ecosystem components alone will not yield the proper values, except potentially in deserts, which have a simple and relatively flat surface. For this reason, numerous studies recur to satellite-based products for ecosystem-scale emissivity. However, this value is based on a directional measurement of a few bands only, and needs to be scaled up to the full spectrum (4-50 µm) by models (Sekertekin and Bonafoni, 2020), while values measured near the ground (e.g., on towers) oftentimes consider the entire spectrum of upwelling diffuse longwave radiation, thus requiring hemispherical emissivity that renders the satellite-based approaches inaccurate. Due to these challenges, a few authors have attempted to obtain plot-scale emissivity using its connection to the plot's sensible heat flux, H (Maes *et al.*, 2019; Thakur *et al.*, 2022). This approach presents its own challenges due to the aerodynamic resistance to sensible heat transfer. Therefore, Maes et al. (Maes *et al.*, 2019) use rare night-time conditions where H is near zero, while Thakur et al. (Thakur *et al.*, 2022) employ an approach based on a linear correlation between the surface-to-air temperature difference and H with a slope and intercept used as indications of the aerodynamic resistance. This second approach, while being more complex, is easier to use in limited datasets since it uses the entire available daytime data.

PV field site: To obtain ecosystem emissivity at our desert and PV sites, we measured samples of the PV panels and of the desert soil beneath the panels and in the surrounding desert using a custom-built direct and high-precision laboratory method (Vishnevetsky *et al.*, 2019). Emissivity values were 0.84 for the PV panels (at an inclination of 30°, since this is the panels' inclination in the PV field), and 0.87 for the soil at 0° inclination (which is considered identical for the shaded soil). Since the PV field is composed by 43% panels and 57% soil, the weighted mean emissivity of the whole field was 0.85 (Table SM2.1).

Desert sites: The methods of Maes et al. (Maes *et al.*, 2019) and Thakur et al. (Thakur *et al.*, 2022) were both used, and lab measurements of different samples (soil and rocks) from the PV field's

desert background site using the method by Vishnevetsky et al. (Vishnevetsky *et al.*, 2019) are shown for comparison (Table SM2.1). At this site, which was completely devoid of vegetation, plot-scale emissivity values calculated using the method by Thakur et al. (Thakur *et al.*, 2022) from the entire dataset and using the method by Maes et al. (Maes *et al.*, 2019) from night-time values agreed. Further, these values agreed with lab measurements, and the value of 0.87 was selected for further calculations.

For the desert adjacent to the Yatir forest, we used data from measurement campaigns north (August 2015 and March 2016) and south (August 2013 and March 2014) of the forest. The southern site was mostly bare soil, while the northern one contained some low shrub cover. For the August 2013 and March 2016 campaigns, the method by Thakur et al. (Thakur *et al.*, 2022) had an intercept of less than 10% of H that is considered acceptable. For the March 2014 and August 2015 datasets, the Maes et al. (Maes *et al.*, 2019) method yielded unreasonable emissivity values larger than 1. Since the other statistical parameters using the method by Thakur et al. (Thakur *et al.*, 2022) were near constant (i.e., slope, RMSE, R^2), we chose the value with the intercept nearest to zero. In the end, the mean between both sites was used for the deserts adjacent to the Yatir forest, i.e. a rounded mean emissivity of 0.88 for summer (August campaigns) and 0.96 for spring (March campaigns). This higher emissivity is a result of annual vegetation in spring.

Forest site: An emissivity of 0.873 was previously calculated for the Yatir forest site by Thakur et al. (Thakur *et al.*, 2022), which was used here. Rock and soil samples from the Yatir forest had a laboratory-measured emissivity of 0.91±0.01 and 0.93±0.01 (mean: 0.92), respectively (Table SM2.1).

Table SM2.1. Emissivity values according to Maes et al. (Maes *et al.*, 2019) and Thakur et al. (Thakur *et al.*, 2022) methods, and the mean of emissivity measurements of soil and rock samples according to Vishnevetsky et al.(Vishnevetsky *et al.*, 2019).

Site	Maes et al. 2019(Maes *et al.*, 2019)	Thakur et al. 2022(Thakur *et al.*, 2022)	Mean of rock and soil measurements
Forest desert background, south (Summer, 2013)	0.864	0.864	

Forest desert background, north (Summer, 2015)	1.002	0.894	
Forest desert background, south (Spring, 2014)	1.09	0.953	
Forest desert background, north (Spring, 2016)	0.96	0.957	
PV background desert	0.868	0.868	0.86
Yatir forest		0.87	0.92
PV field			0.85

Methods S3: Additional climate zones

The break-even time calculation (Methods) was performed for additional two locations as a comparison to dryland sites: a tropical rainforest, combining data from Panama (PA-SPn for forest, and PA-SPs for the grassland) and the Amazon forest, and a temperate zone using data from Germany (DE-Hzd for the forest, and DE-Gri for the grassland). Instead of a desert, the grassland is the background either for forests or PV fields. Therefore, we tried to find PV fields with nearby grassland and forest Eddy Flux sites. Albedo and NEE data for tropical sites and NEE for the temperate zone were obtained from the literature (Luyssaert *et al.*, 2007; SPA, 2021), and albedo of the temperate zone from the Euroflux database (http://www.europe-fluxdata.eu/). Solar irradiance (Eg, W m^{-2}) and thermal radiation emission (L_{out}, W m^{-2}) data in the tropical zone was downloaded from Fluxnet (https://fluxnet.org/), considering the range of years from 2015-2021 for the temperate and 2007-2009 for the tropical regions, depending on data availability.

The land-use efficiency of the PV field was based on Eg and PV field production (Annual Electricity Production, EPa kWh m^{-2} yr^{-1}). EPa is obtained for the total yearly PV field production data (GPPD, 2018) divided by the entire surface area of the field (including areas between panels and field infra-structure, measured using Google Earth). EPA was 100 kWh m^{-2} yr^{-1} for the temperate and 130 kWh m^{2} yr^{-1} for the tropical zones. PV_{eff} (0.11 for the temperate and 0.09 for the tropical zones) is the result of EPa divided by the mean annual irradiation (900 kWh yr^{-1} for the temperate and 1500 kWh yr^{-1} for the tropical zones) (Kawajiri, Oozeki and Genchi, 2011). Eg was calculated from the mean yearly incoming shortwave radiation (S_{in}, W m^{-2}) at each of the close by Eddy flux site.

The albedo of forests and grasslands in Germany was calculated based on available up- (S_{out}) and downwelling shortwave radiation measurements (S_{in}). The mean albedo at each site was considered to be the median of yearly albedo values. Yearly albedos were obtained as the mean daily albedo weighted by the mean daily S_{in}. Daily albedo values were obtained from the daily sums of half-hourly S_{in} and S_{out} measurements (Equation SM3.1).

$$\alpha_{yearly} = \frac{\alpha_{daily}\, S_{in,daily}}{\sum S_{in,daily}}$$

(SM3.1)

$$\alpha_{daily} = \frac{S_{out,daily}}{S_{in,daily}} = \frac{\sum S_{out,30min}}{\sum S_{in,30min}}$$

The thermal radiation emission suppression of forests was calculated as the difference in yearly mean upwelling longwave radiation (L_{out}) between grassland and forest sites. As was missing at tropical sites, it was calculated from S_{in}, L_{in} and R_n (Equation SM3.2). L_{out} differences were not significant ($p > 0.5$) in both these climate zones, and therefore, considered to be 0 in the radiative forcing (RF_r) calculation.

(SM3.2) $$L_{out} = S_{in} (1 - \alpha) + L_{in} - R_n$$

The albedo of the PV fields was calculated from the albedo of PV panels (Burg *et al.*, 2015) (0.05) and that of background grasslands (Equation SM3.3):

(SM3.3) $$\alpha_{PV} = (\alpha_{panel} \cdot \%_{panel}) + (\alpha_{grass} \cdot \%_{grass})$$

The percentage of land cover of each was estimated from Google Earth by measuring the width of PV panels and of the area in between, rounding down the area of panels to account for other field infra-structure (Figure SM3.1). The temperate PV field (Germany – Oberrossau, GPPD identifier = WKS0062541) area occupied by rows of panels intercalated by background grassland corresponds to 90% of the field, while the other 10% is composed by background grassland with some infrastructure (roads). Thus, the resulting total coverage is 45% PV panels and 55% background grassland considering the whole field area. In the lower latitudes of the tropical PV field (Panama – Divisa Solar, GPPD identifier = WKS0065072), the PV panels rows are closer together, and compose *ca.* 60% of the total area of the field, after accounting for the necessary open spaces and infrastructure.

Table SM3.1. Selected PV fields in the temperate (Germany - Oberrossau) and tropical (Panama – Divisa Solar) zones. The identifier follows the GPPD v. 1.3 database classification. The percentage of panels was estimated from Google Earth, and the albedo of the PV field was calculated using Equation SM3.1. The estimated electricity production by the PV field (EP, kWh year^{-1}) was obtained from the GPPD v. 1.3 database. The PV fields area (m^2) was estimated from Google Earth using QGIS software. EPa (kWh m^{-2} yr^{-1}) was calculated using Equation M5. The efficiency of the PV field (PV_{eff}) was calculated as PV_{eff} = EPa/Eg.

	**Temperate**	**Tropical**
GPPD Identifier	WKS0062541	WKS0065072
Coordinates	50.987°N, 13.074°E	8.185°N 80.711°W
% of panels	45	60

$\alpha_{PV\ field}$	0.13	0.13
$\Delta\alpha_{background\ –\ PV\ field}$	0.07	0.08
EP	10	17
Area	110,000	130,000
EP_a	100	130
PV_{eff}	0.11	0.09

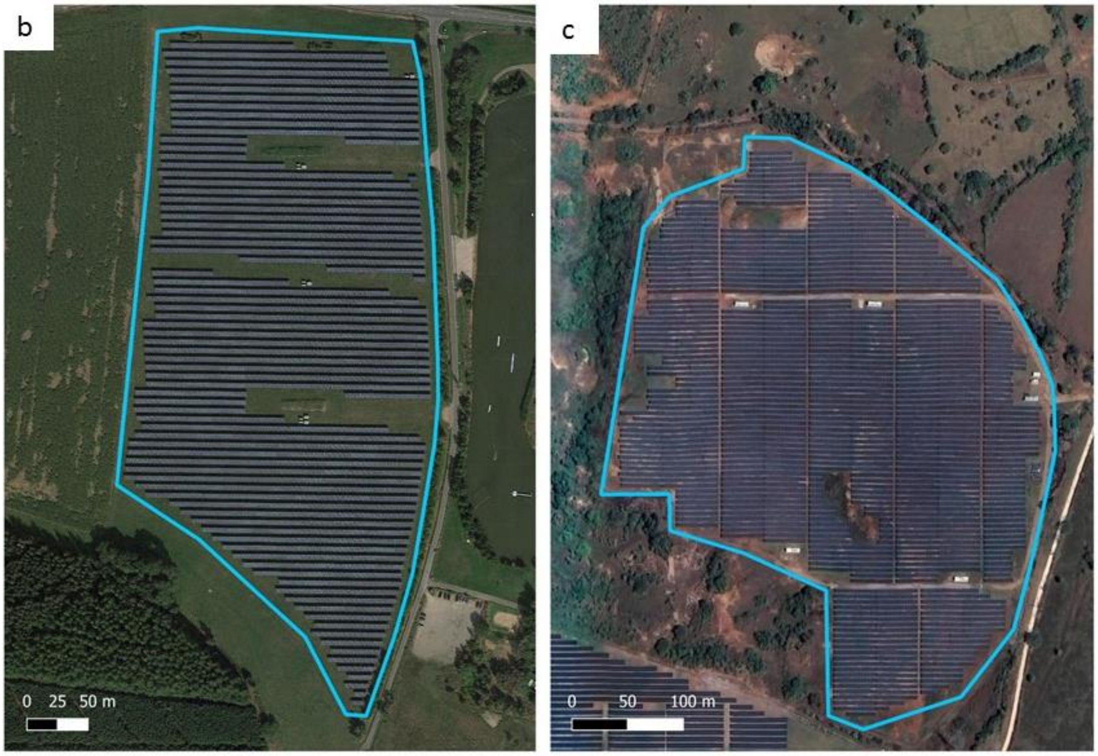

Figure SM3.1. Map of the area of the temperate (a) and tropical (b) PV fields as measured from Google Earth, showing the area of panel rows and infrastructure of the fields.

Methods S4: Thermal radiation and surface temperature using UAV and mast measurements

Ecosystem longwave radiation and surface temperatures: The ecosystem-scale upwelling thermal longwave (L_{out}) was directly obtained from measurements. The ecosystem surface temperatures (T_s, in K) were obtained by reversing the Stefan-Boltzmann equation:

(SM4.1) $$T_s = \sqrt[4]{\frac{L_{em}}{\varepsilon\sigma}}$$

Where L_{em} is the emitted longwave radiation by the surface, ε its emissivity (Methods S2) and σ the Stefan-Boltzmann constant. L_{em} is a function of L_{out}, corrected for the reflected downwelling thermal radiation from the atmosphere (L_{in}), measured on the mast:

(SM4.2) $$L_{em} = L_{out} - (1 - \varepsilon)L_{in}$$

Unmanned aerial vehicle (UAV) measurements in the PV field: Measurements of upwelling radiation from mixed surfaces (such as PV fields or ecosystems) are typically obtained from down-looking radiation sensors at a height that captures a proper representation of the different surfaces and their characteristics inside the viewing angle of the sensors. In the spring campaign, measurements were therefore performed between the lines of panels at a height of 5.2 m in a position which allowed a proper representation of the field elements (PV panels, rows of sun-lit and shaded soil; Figure S4). However, in subsequent campaigns (summer and autumn), due to shading impact on the panel performance and the potential damage of single cells due to the resulting under-voltage, we had to move the measurements to an operational space between the rows. The sensors were deployed at a height of 4.5 m above ground, which was enough for the eddy-covariance measurements (Figures S4 and S5), but insufficient to capture the field heterogeneity by the radiation sensors. Therefore, we combined these mast measurements with an innovative approach using UAV (*Unmanned Aerial Vehicle*; DJI Matrice 200, Shenzhen DJI Technology Ltd., Shenzhen, China; Figure SM4.1) equipped with a thermal camera (DJI Zenmuse XT2, RGB and thermal range: 7.5 -1 3.5µm, 640×512 pixels resolution) in order to correct the mast measurements to a proper PV field representation.

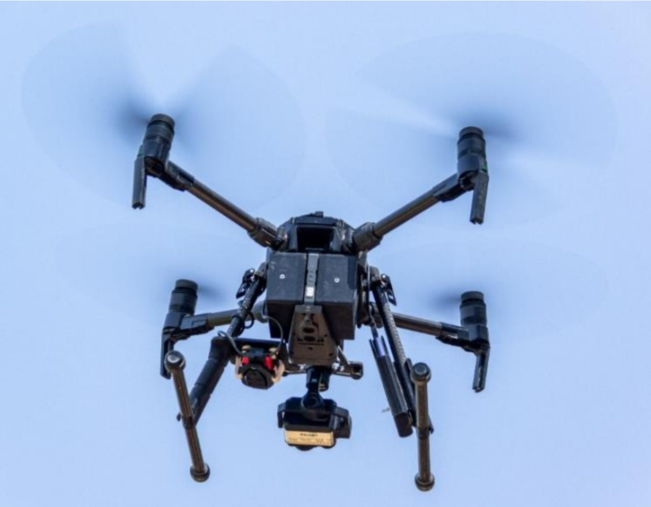

Figure SM4.1. Research UAV equipped with multi-spectral and thermal cameras.

In the PV field, multiple flights were carried out daily in the period between 06:00 and 20:00 (*ca.* every 1.5-2h), and covered an area approximately corresponding to the eddy-covariance fetch (*ca.* 50×50m; Figure S5). Each flight was done at a height of 50m above ground with a duration of *ca.* 5 min and provided around 100 images. At the desert background site, the eddy-covariance mobile station mast height of 4.5m was sufficient to cover a representative area of the more homogenic desert surface. Therefore, a few UAV flights were performed in that area and their IR images were used to calibrate the mast's radiation measurements of the PV field to values equivalent to those of a mast at the appropriate height.

T_s and L_{out} of the entire PV field: The surface temperature of the entire PV field was obtained from corrected continuous upwelling thermal radiation dataset measured in the PV field ($L_{out,mobile,cor}$), according to SM 4.1 and SM 4.2. As the mobile system's radiation sensors viewing footprint had to be corrected in order to represent the field heterogeneity in the right proportion, its measurements ($L_{out,mobile}$) were corrected using a linear calibration curve based on UAV flights:

(SM 4.3)

$$L_{out,mobile,cor} = a\ L_{out,mobile} + b$$

The parameters *a* and *b* were obtained from a linear correlation (Figure SM4.2) between calibrated UAV-based thermal camera measurements of the PV field ($L_{out,UAV,cal}$) and of the sensors at the mobile mast ($L_{out,mobile}$), combining both seasons.

(SM 4.4)

$$L_{out,UAV,cal} = a\ L_{out,mobile} + b$$

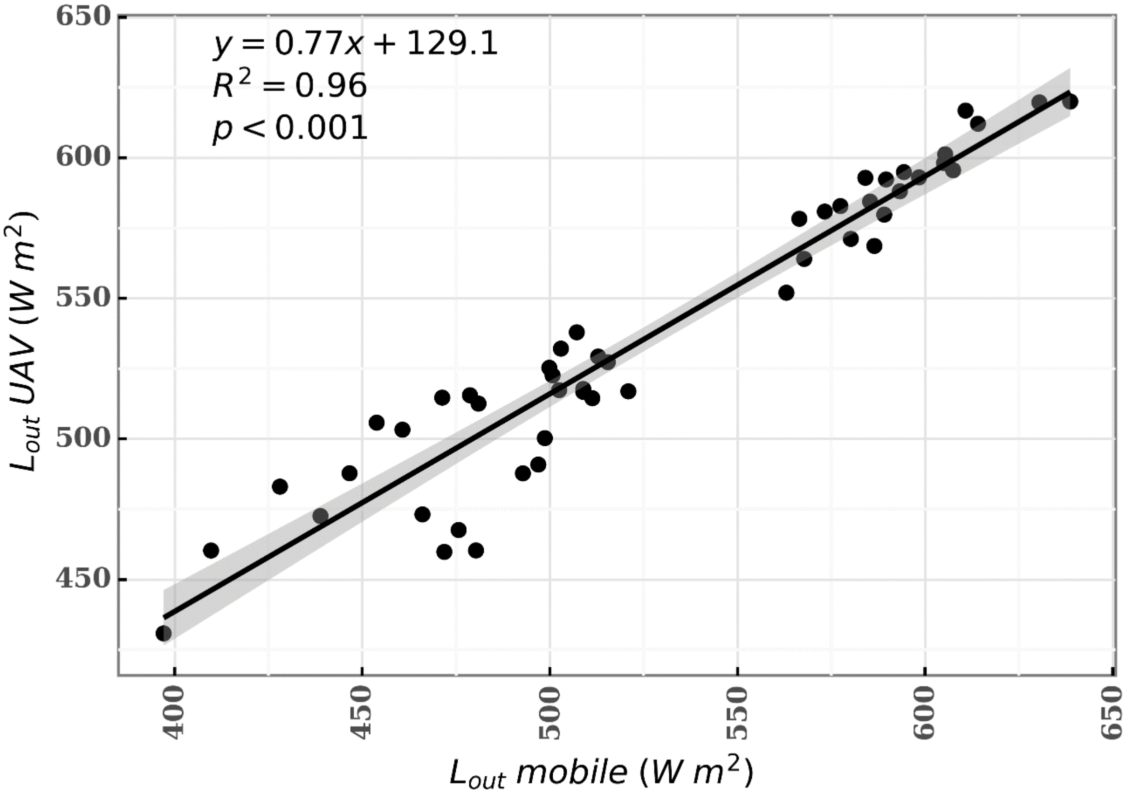

Figure SM4.2. Linear correlation between the thermal radiation measurements of the mobile system's CGR-4 mast-mounted sensors ($L_{out,mobile}$) and the UAV's thermal camera ($L_{out,UAV}$) during the autumn and summer campaigns in the PV field.

The UAV measurements over the PV field were adjusted according to calibration parameters determined from calibration flights in the background desert, where the mobile system-based sensors measured a representative area.

(SM 4.5) $$L_{out,UAV,cal} = c\, L_{out,UAV} + d$$

The calibration resulting in parameters *c* and *d* was necessary due to sensor differences between the mast and UAV thermal radiation measurements, such as spectral range (Mast CGR-4 sensor: 4.5 – 42 µm; UAV: 8 – 14 µm) and viewing angles (CGR-4, 180°, UAV 26° - 32°). This calibration was performed using the combined data of 2 flights in autumn and 3 flights in summer (Figure SM4.3).

(SM 4.6) $$L_{out,mobile} = c\, L_{out,UAV} + d$$

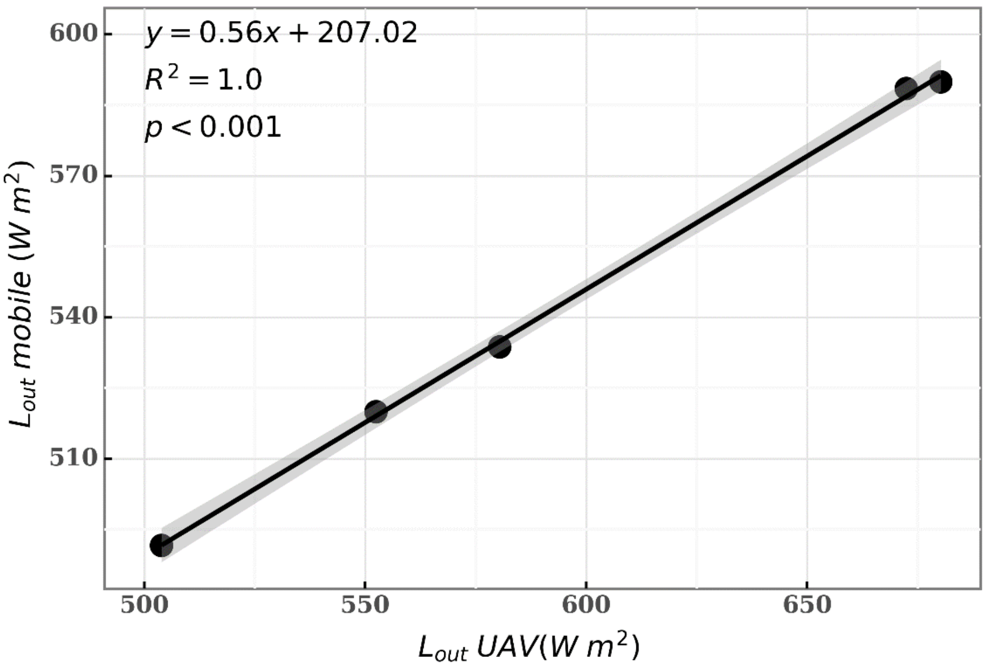

Figure SM4.3. Linear correlation between thermal radiation measurements by the UAV's thermal camera ($L_{out,UAV}$) and the mobile system's mast-mounted sensors ($L_{out,mobile}$) during the autumn and summer campaigns in the desert background.

The upwelling radiation of the surface, measured by the UAV's infrared camera ($L_{out,UAV}$), was considered to be the mean of all pixels of all IR images taken during each flight. These IR images provide an apparent temperature (T_{ap}), which corresponds to the temperature measured by the infrared camera using an emissivity setting of 1, from which $L_{out,UAV}$ is calculated:

(SM 4.7) $$L_{out,UAV} = 1\sigma T_{ap}^4$$

T_s and L_{em} of each PV field category: Since thermal infrared cameras calculate an 'apparent' temperature of each pixel (T_{ap}) based on a fixed emissivity setting applied to the entire image (here: 1), temperatures of each pixel must be corrected using their correct ε and reflected L_{in} for each pixel. Therefore, we developed an algorithm based on deep learning (Falk *et al.*, 2019) using Python 3.6 with the "OpenCV" library (Bradski, 2000) in order to segment the IR images of the PV field into 3 categories: panels, sun-exposed soil and shaded soil (Figure S7). Using this segmentation, both the fractions of each category in the entire PV field, their temperature (Discussion S1) and the mean emitted thermal radiation were calculated.

The mean surface temperature (T_s) of each category was calculated from the mean emitted thermal radiation (L_{em}) according to Equation SM4.8. L_{em} was calculated by first accounting for background effects described in the infrared camera equation (Incropera *et al.*, 1996), and then averaging all the pixels of each category and flight.

(SM4.8) $$L_{em} = \frac{\sigma T_{ap}^{4} - \tau\,(1-\varepsilon)\,L_{in} - (1-\tau)\,L_{air}}{\tau\,\varepsilon}$$

Where ε is the emissivity of each category and L_{air} is the longwave radiation emitted by the air column between the object and the camera. This last term is dropped since the transmissivity of the air between the object and the camera (τ) is considered to be 1 due to the dry air and short distance of ca. 50m between the IR camera of the UAV and the object.

Methods S5: Shade albedo

Normally, albedo is defined as upwelling (S_{out}) divided by downwelling (S_{in}) radiation, and in shaded conditions, the same is true for up- ($S_{out,dif}$) and downwelling diffuse radiation ($S_{in,dif}$).

(SM5.1) $$\alpha = \frac{S_{out}}{S_{in}} = \frac{S_{out,dif}}{S_{in,dif}}$$

However, for sensors deployed in a nadir-view perspective, the albedo of shaded soil (α_{sh}) is related to the direct downwelling solar radiation (S_{in}), while the upwelling part is that of the shade ($S_{out,dif}$).

(SM5.2) $$\alpha_{sh} = \frac{S_{out,dif}}{S_{in}}$$

Since the albedo of exposed ground is known, the albedo of shade can be related to the diffuse fraction of the downwelling radiation ($f_{dif} = S_{in,dif}/S_{in}$), and therefore to the albedo of exposed soil:

(SM5.3) $$\alpha_{sh} = \frac{S_{out} \cdot f_{dif}}{S_{in}} = \alpha \cdot f_{dif}$$

Methods S6: PV panel efficiency

The current study is focused on the land efficiency of PV installations (PV_{eff}) with regard to climate change mitigation through carbon emission suppression. However, this efficiency is different from PV panel efficiency ($PV_{eff,panels}$), which is a function of electricity production and insolation. Note that the calculation of $PV_{eff,panels}$ is provided here as only a validation of the soundness of our comparisons between climate zones (temperate, dryland and tropical), but its optimization is not part of the current study.

While the PV panel surface area (A_{panels}; a function of the length of a panel, L_{panels}) exposed to solar radiation may be the same at different latitudes, the projected PV panel area on the ground ($A_{panels,proj}$; a function of the projected length, l_{panels}) differs significantly due to their inclination angle (γ; Figure SM6.1). This inclination angle is typically optimal when it corresponds to the latitude, which is our assumption in the current calculation. As a result, PV panels could potentially be arranged more densely at higher latitudes (i.e., the number of panels per land surface area), which would increase PV_{eff}. However, this may make them more prone to shading each other during winter time. Still, in an annual mean, EP_a could be still high when the panels inclination is optimized for the summer months. Nevertheless, PV_{eff} is higher in a temperate climate such as Germany's (as shown in Table 3).

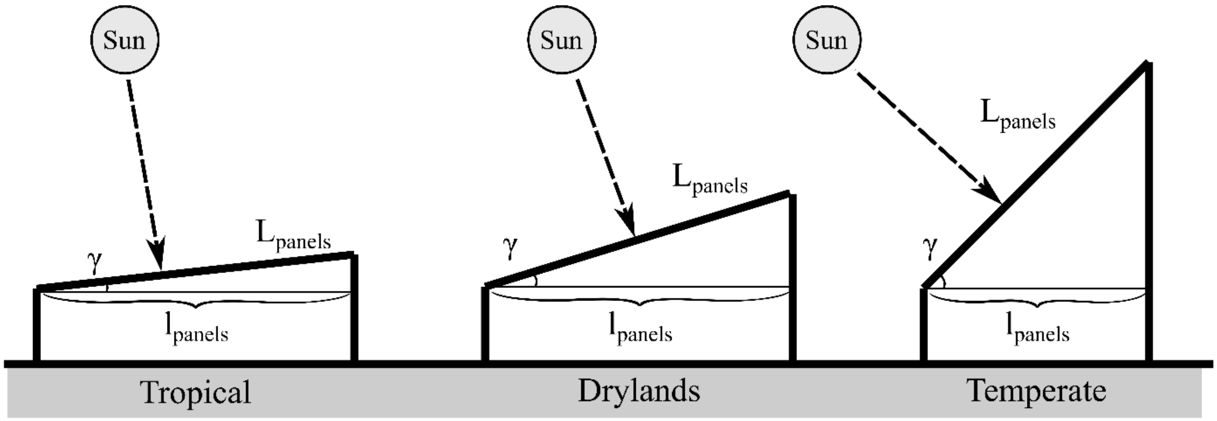

Figure SM6.1. Projected (nadir) land surface area (l_{panels}) differences between the tropical, dryland and temperature scenarios differing in spite of the same effective PV panel surface area (L_{panels}), as a result of the inclination angle of PV panels (γ) optimized for the latitude, i.e. the solar angle

at noon at equinox. For the same panel surface area (L_{panels}), the land surface area (l_{panels}) is larger in the tropical than in the temperate climate, where more rows can be placed together.

Panel efficiency ($PV_{eff,panels}$) is defined similarly to PV_{eff}, but using the surface area of PV panels, as a function of annual electricity production of a PV field per its land area ($EP_{a,panels}$; kWh m^{-2} yr^{-1}) and the annual insolation (Eg; kWh m^{-2} yr^{-1}):

(SM6.1) $$PV_{eff,panels} = \frac{EP_{a,panels}}{Eg}$$

$EP_{a,panels}$ corresponds to the electricity production of the field (EP) divided by its panel surface area (A_{panels}):

(SM6.2) $$EP_{a,panels} = \frac{EP}{A_{panels}}$$

Where A_{panels} is simply a function of the projected panel area ($A_{panels,proj}$) and their inclination angle (γ), corresponding to the latitude:

(SM6.3) $$A_{panels} = \frac{A_{panels,proj}}{\cos\gamma}$$

Finally, $A_{panels,proj}$ is a function of the measured fraction of panels ($f_{panels,proj}$) on a PV field and the field's total measured surface area (A_{field})

(SM6.4) $$A_{panels,proj} = f_{panels,proj}\, A_{field}$$

Note that the resulting panel efficiencies between the temperate, dryland and tropical climates did not significantly differ using the calculation above (Table SM6.1). Small differences may be due to different PV panel technologies or temperature differences of sub-optimal installations (e.g. inclination angle).

Table SM6.1. PV panel efficiency ($PV_{eff,panels}$) across latitudes in the temperate (Germany - Oberrossau) dryland (Israel - Ketura) and tropical (Panama – Divisa Solar) zones.

Climate zone	Latitude (deg north)	$PV_{eff,panels}$
Tropical	9	0.14
Dryland	30	0.12

Temperate	51	0.15

Supplementary Discussion

Discussion S1: Temperature

The air temperature (5 meters above ground level) over the PV field was significantly lower than over the desert in autumn (difference of 3.4°C, $p < .001$, note that a rare rain storm occurred in the beginning of the campaign over the PV field, which seemingly contributed to this large temperature difference) and spring (difference of 1.2°C, $p < .05$). In summer, the air temperature over the PV field was 0.9 °C lower than that over the desert background ($p < .01$, Table SD1.1).

Table SD1.1. Daily (24 hours) means and standard deviations (in parenthesis) of air temperature (°C) in both the desert background and the PV field, in all three measurement campaigns.

T_a			
Season	Desert Background	PV field	p
Summer	34.5 (4.7)	33.4 (4.1)	< .01
Autumn	28.2 (3.5)	24.8 (4.0)	< .001
Spring	26.0 (4.5)	24.8 (5.3)	< .05

An analysis of the diurnal cycle of air temperature (Figure SD1.1) reveals that in autumn, the air temperature is uniformly lower over the PV field than over the desert background during day and night ($p < .001$). The differences were also significant during summer ($p < .001$). In spring, the difference was not significant ($p = 0$).

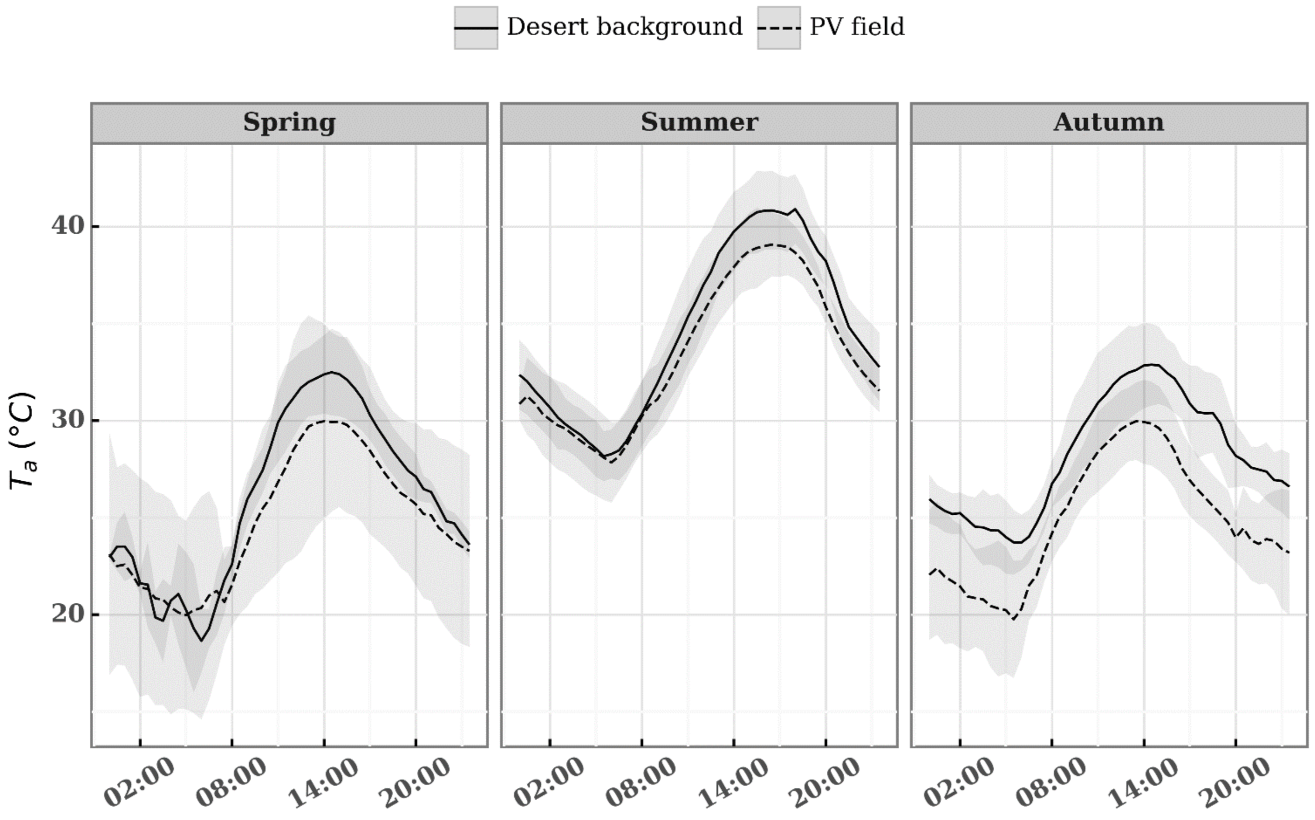

Figure SD1.1. Diurnal cycles of half-hourly means and standard deviations of air temperature for both the desert background and the PV field during the spring (left), summer (center) and autumn (right) campaigns. Solid and dashed lines represent the mean values for the desert background and PV field, respectively. The shaded area around the lines represents one standard deviation from the means.

T_s, derived from the thermal radiation measurements obtained by the UAV for each element of the PV field (panels, sun-exposed soil and shaded soil; cf. Methods S4) are presented in Figure SD1.2. The PV panels had the highest temperatures, and the shaded soil the lowest, while the sun-exposed soil temperatures were intermediate (Figure SD1.2, $p < .001$ for all the differences). At midday (10:00 – 15:00), mean temperatures of the panels were up to 15°C higher than the shaded soil below it, and 8°C higher than the exposed soil.

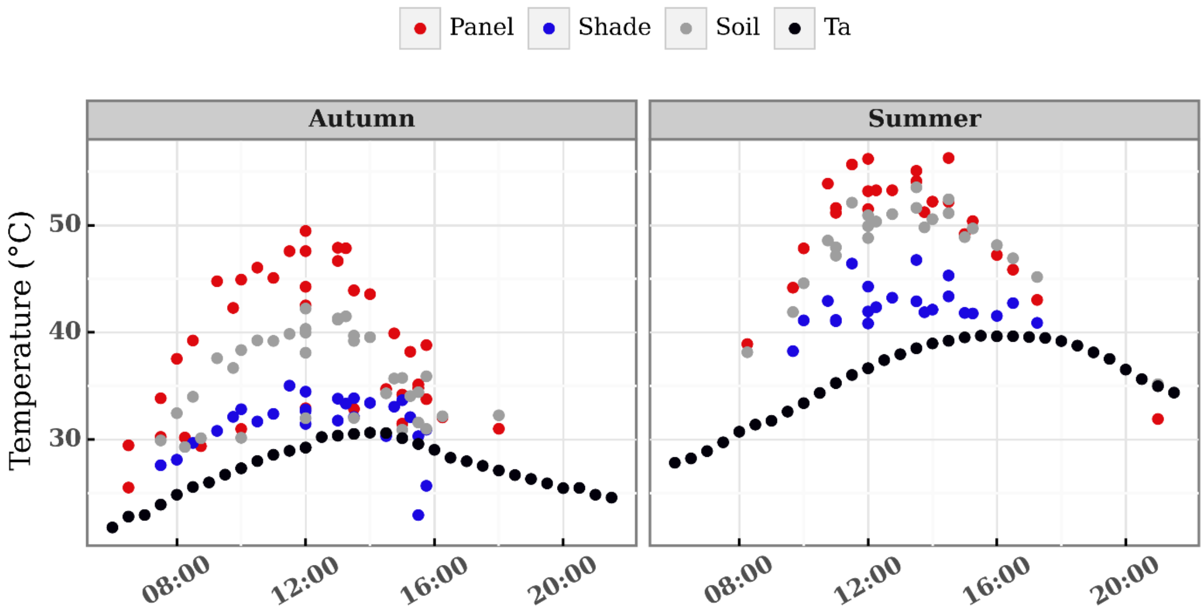

Figure SD1.2. Temperature of the different elements of the PV field estimated from the UAV images analysis, during the autumn campaign (left), and the summer (right).

Discussion S2: Radiative and non-radiative fluxes

Upwelling thermal radiation (L_{out}): L_{out}, separated by each element of the PV field, is presented in Table SD2.1, based on the segmentation analysis. It reveals that the PV panels are the element with the highest L_{out}, followed by the sun-exposed soil. The standard deviation of the L_{out} values measured in the shadow during the autumn were much higher than in any other element in both seasons. In the summer, differences were significant ($p < .001$, and $p < .05$ in the comparison between panels and sun-exposed soil) in the comparison between all the elements, while in the autumn differences were significant ($p < .001$) in most cases, besides between panels and sun-exposed soil ($p > .05$). Figure SD2.1 shows the diurnal cycles of L_{out} in each season, which were not significant in the spring and autumn (Table SD2.2, $p > .05$), and significant during summer (Table SD2.2, $p < .05$), when night-time L_{out} was consistently higher in the PV field ($p < .001$ during night).

Table SD2.1. Mid-day means (10:00 – 15:00) and standard deviations (in parenthesis) of the outgoing longwave radiation (L_{out}) of the three elements of the PV field (panel, shade and soil), measured with the UAV during the summer and autumn campaigns. Values are expressed in units of W m^{-2}.

L_{out}			
Season	Panel	Shade	Soil
Summer	539 (15)	493 (12)	538 (15)
Autumn	475 (34)	397 (86)	463 (21)

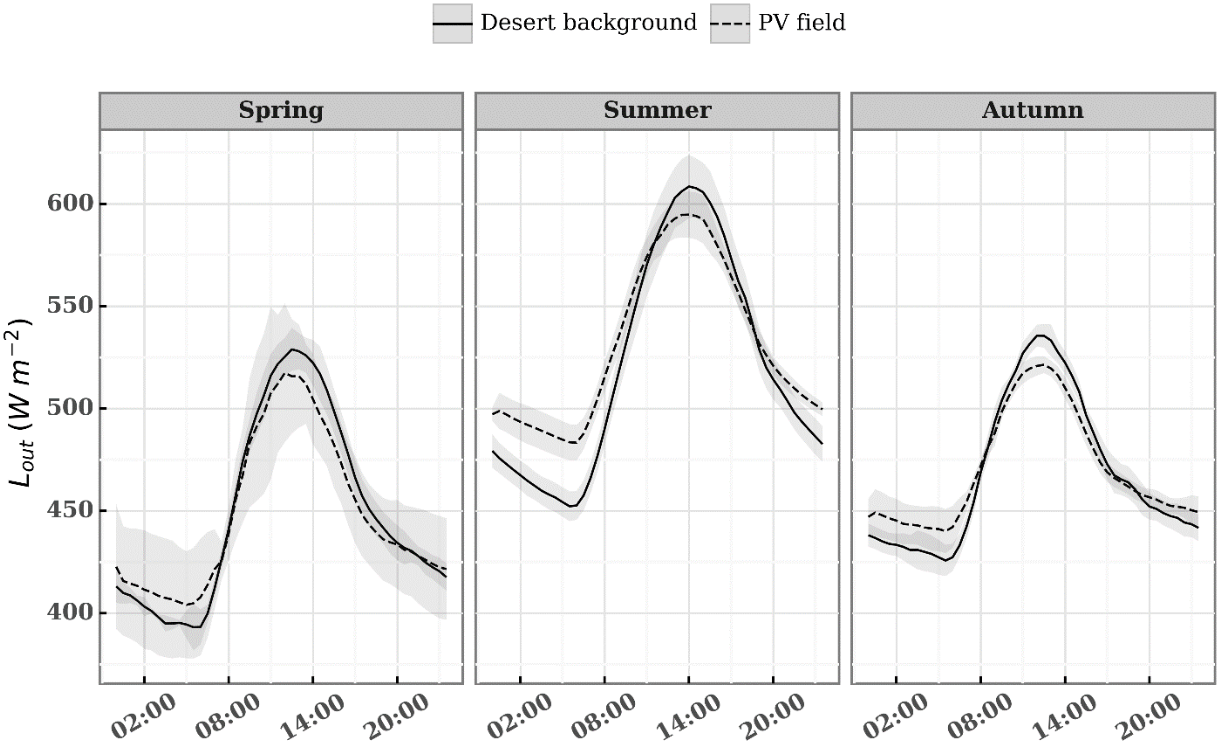

Figure SD2.1. Diurnal patterns of the L_{out} for both the desert background and the PV field in the spring (left), summer (center) and autumn (right) campaigns. Solid lines represent the mean values for the desert background, and the dashed lines represent means for the PV field. Each half-hour interval represents the mean of all the same half-hour interval of every day of measurements of each field and season. Shaded area around the lines represents one standard deviation from the means.

Downwelling radiative fluxes: Downwelling radiative fluxes are presented for each season. L_{in} presented significant differences (Table SD2.2, $p < .001$) between the desert and the PV field during all the seasons. While L_{in} was larger in the PV field during spring and summer, it was larger in the desert during the autumn, due to a rare rain event during the PV field campaign, which decreased air temperature (Discussion S1). Diurnal patterns of L_{in} reveal consistent differences between day and night (Figure SD2.2), which were significant in the autumn ($p < .001$) and summer ($p < .001$ during day and $p < .01$ during night), but not in the spring.

As expected, differences in S_{in} were not significant between both locations and in every season, since it depends on insolation intensity (Table SD2.2). However, differences in S_{out} were

significant ($p < .001$) between the desert and the PV field in all seasons (Table SD2.2). This result was expected, since it reveals the albedo effect.

Table SD2.2. Mid-day (10:00 – 15:00) means and standard deviations (in parenthesis) of the radiative fluxes in the desert background and the PV panels field, where p values denote the significance of difference between the means of the comparison between desert background and PV field. Units are W m^{-2}. Note that L_{in} was lower in the PV field than in the desert background in Autumn due to a cooler atmosphere after a rare rain event.

	Desert background	PV field	p
Autumn			
S_{in}	640 (122)	654 (126)	0.9
S_{out}	244 (46)	89 (15)	<.001
L_{in}	390 (9)	349 (10)	<.001
L_{out}	526 (10)	515 (7)	0.52
Spring			
S_{in}	854 (99)	769 (136)	0.43
S_{out}	337 (40)	119 (21)	<.01
L_{in}	346 (13)	356 (19)	<.001
L_{out}	519 (13)	507 (29)	0.71
Summer			
S_{in}	965 (68)	945 (71)	0.44
S_{out}	361 (25)	180 (11)	<.05
L_{in}	392 (13)	402 (15)	<.001
L_{out}	586 (26)	583 (16)	<.05

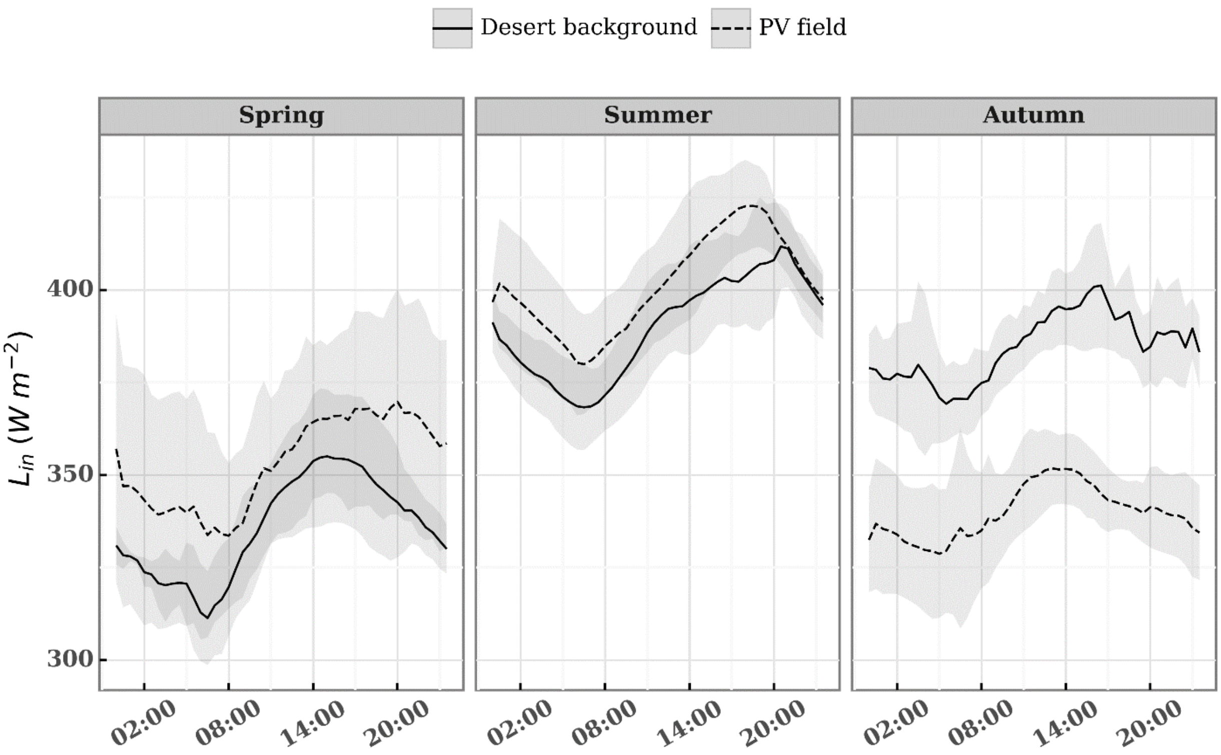

Figure SD2.2. Diurnal patterns of the L_{in} for both the desert background and the PV field in the spring (left), summer (center) and autumn (right) campaigns. Solid lines represent the mean values for the desert background, and the dashed lines represent means for the PV field. Each half-hour interval represents the mean of all the same half-hour interval of all the measurements days of each field and season. Shaded area around the lines represents one standard deviation from the means. Note that L_{in} was lower in the PV field than in the desert background in Autumn due to a cooler atmosphere after a rare rain event

Non-radiative fluxes: Since the PV field is located in a hyper-arid region, LE fluxes have a very small participation in the dissipation of the radiative energy, with highest mid-day (10:00 – 15:00) mean values of around 35 W m^{-2} during the summer (Table 1, Figure SD2.3), and lowest around 14 W m^{-2} during the spring (Figure SD2.3), and no significant variations between the desert and the PV field. As a result, the most important non-radiative energy flux is H (Figure SD2.3). The mid-day mean of H in the summer was 198 W m^{-2} in the desert background and about 80% higher with 358 W m^{-2} in the PV field ($p < .001$, Table 1, Figure SD2.3). In the autumn it was 144 W m^{-2} in the desert and 74% higher with 251 W m^{-2} in the PV field ($p <.001$). During the spring, the difference in H between the desert and the PV field was not significant ($p > .05$), with 173 W m^{-2}

in the desert and 178 W m^{-2} in the PV field. R_n differences were significant ($p < .001$) only in the summer, with 409 W m^{-2} in the desert and 529 W m^{-2}. PV_e is only present in the PV field, and the mid-day mean was highest in the summer (54 W m^{-2}), and lowest in the autumn (37 W m^{-2}). The half-hourly energy output of solar panels (PV_e, W m^{-2}) was obtained by multiplying PV_{eff} (5.8%) by S_{in} (W m^{-2}).

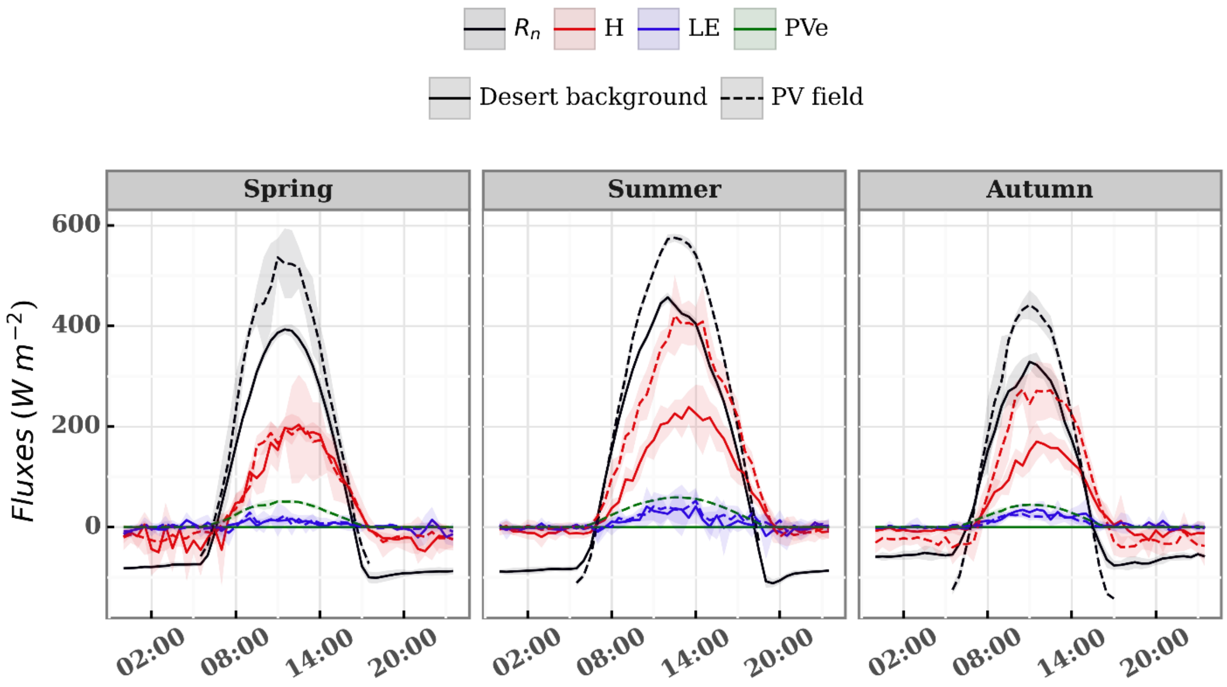

Figure SD2.3. Diurnal cycles of campaign means and standard deviations (shaded area) of the fluxes of H (red), LE (blue) and Rn (black) for both the desert background (solid lines) and the PV field (dashed lines), in the spring (left), summer (center) and autumn (right). The electricity generated by the PV panels (PV_e) is showed in green. Each half-hour interval represents the mean of all the same half-hour interval of every day of measurements of each field and season.

Supplementary Figures

Figure S1: Experimental arid measurement sites in an afforestation system

a) desert adjacent to the forest; b) Yatir forest; c) desert adjacent to the PV field; d) PV field; e) their locations in the Negev Desert, Israel (Google Earth, TerraMetrics & GISrael 2022) and f) global aridity index map, based on aridity index classification by (Trabucco and Zomer, 2018), where it is possible to see that our research sites are under the same classification as most of global drylands.

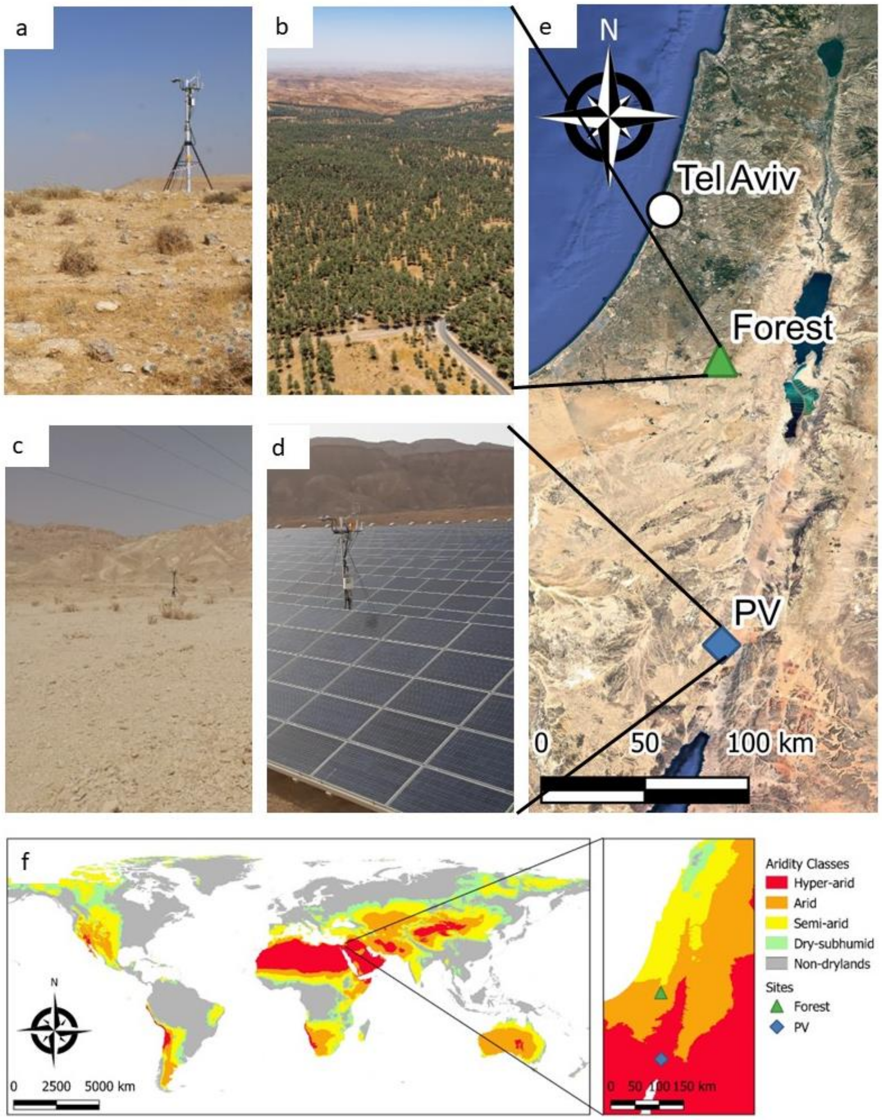

Figure S2: Meteorological conditions during measurement campaigns

Comparison between daily means of meteorological conditions (grey) and measurement campaigns (blue) for incoming shortwave radiation (S_{in}, W m^{-2}) and air temperature (T_a, °C). Long-term meteorological data were obtained from the Israeli Meteorological Service for the PV field area (Yotvata station) and the above-canopy measurements at the Yatir forest eddy covariance site. The campaigns represent the overall meteorological conditions.

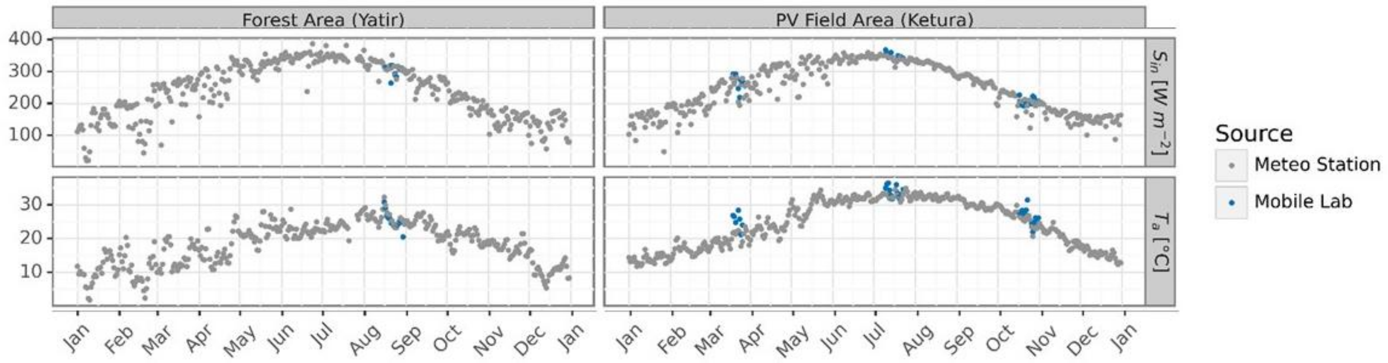

Figure S3: Area considered for the PV field

Area considered for the PV field, including the rows of panels and soil (84% of the field), as well as roads and infrastructure (16% of the field). The middle road is 7m wide, and therefore a margin of 7m was considered in the whole perimeter of the panels rows (blue line), considering the minimum space needed for the field operation. Considering all these land-use needs, it is possible to estimate the minimum area occupied by the PV field for generating electricity (75,600 m^2 in this case).

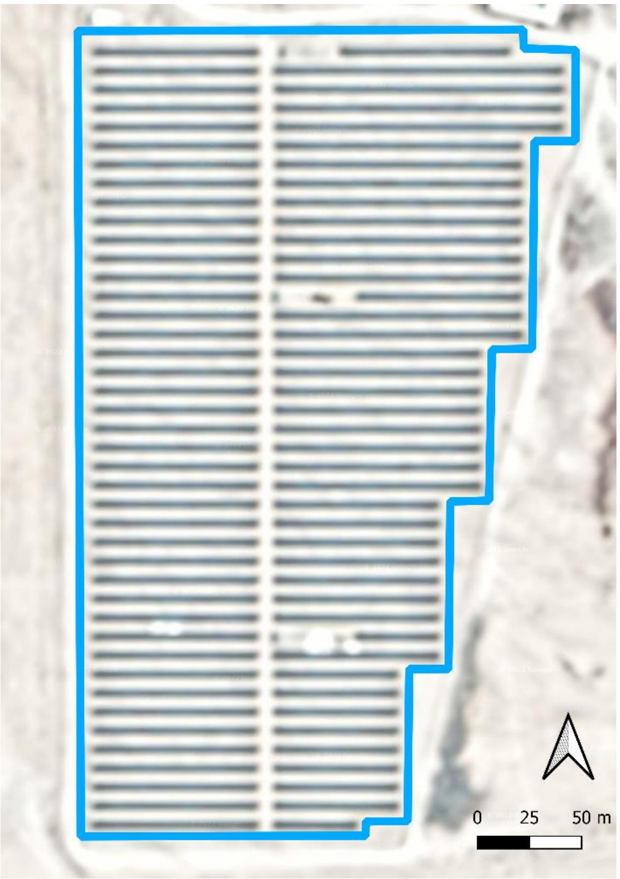

Figure S4: Mobile Eddy-Covariance System

a) Mobile system with eddy-covariance, radiation and meteorological sensors in the field, b) measurements mast in the desert background, c) in the PV field and d) a view from above of the PV field taken with an UAV (unmanned aerial vehicle), in which the mast location is marked with a red star.

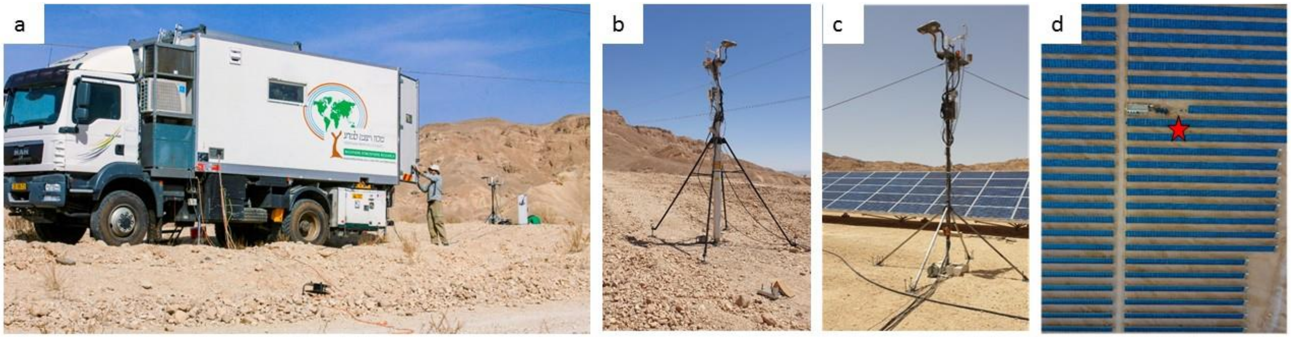

Figure S5: Footprint analyses of measurement campaigns

Footprint analyses of measurement campaigns in the desert background (top) and in the PV field (bottom) using the Tovi™ software (LI-COR Biosciences; Lincoln, NE, USA), showing that >80% of the fluxes in every measurement campaign originated from areas inside the fetch of interest. During the spring campaign in the PV field, the mast was placed on the edge of the field due to constraints from the field operator, but the majority of the fluxes also came from the PV field and the remaining ones were filtered out. In the following campaigns, it was possible to place the mast in a more central location in the field, thus avoiding a possible mixture of fluxes from outside the area of interest.

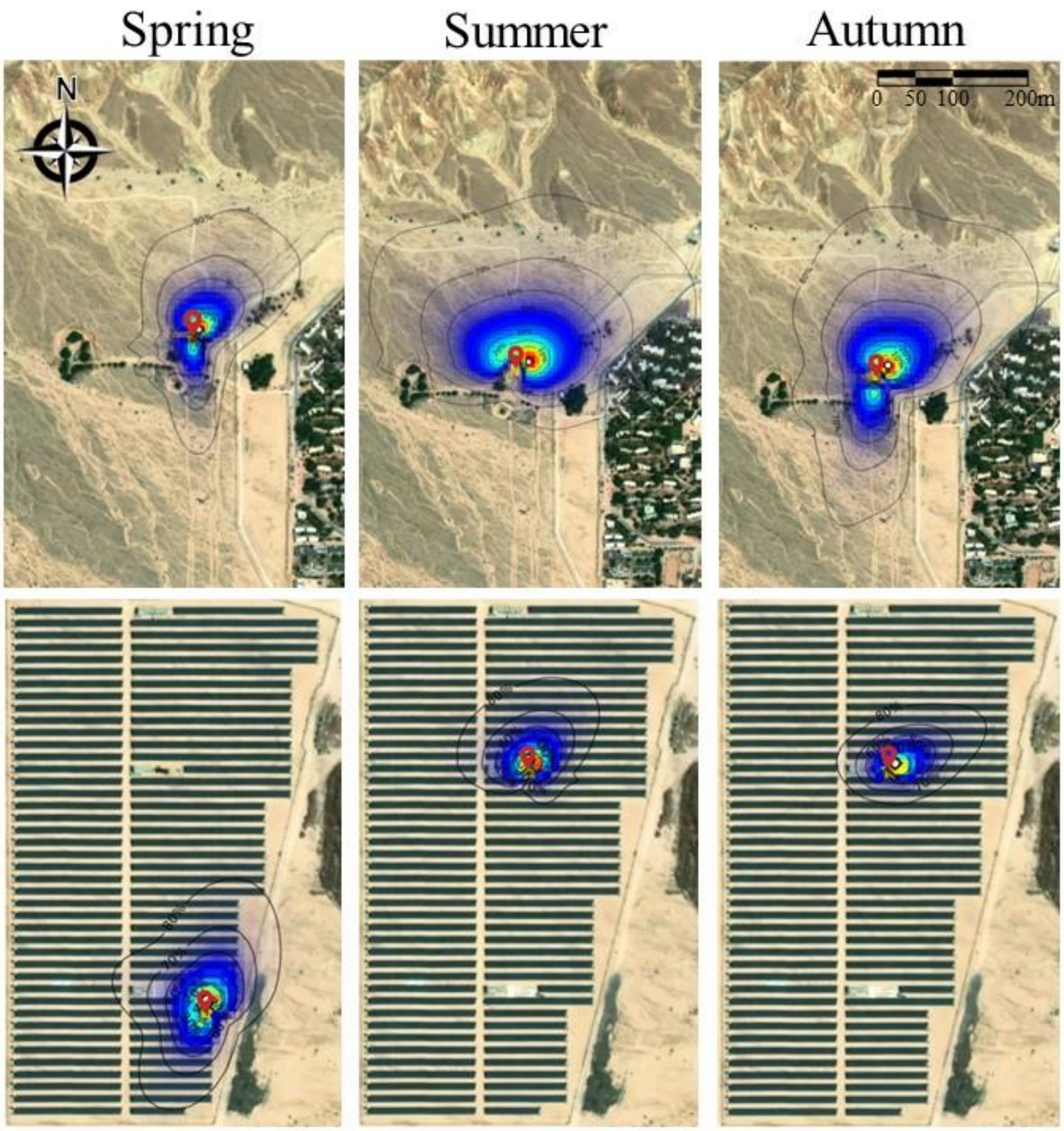

Figure S6: Wind roses of the mean shear velocity (u*, turbulence) and wind direction

Wind roses of the mean shear velocity (u*, turbulence) and wind direction in (a) the desert background and in (b) the PV field during each season's measurement campaign. Colors represent the shear velocity (m s^{-1}), and the length represent the frequency of occurrence. Only a slight increase in u* of ca. 0.2 m s^{-1} was observed over PV fields during autumn and summer campaigns.

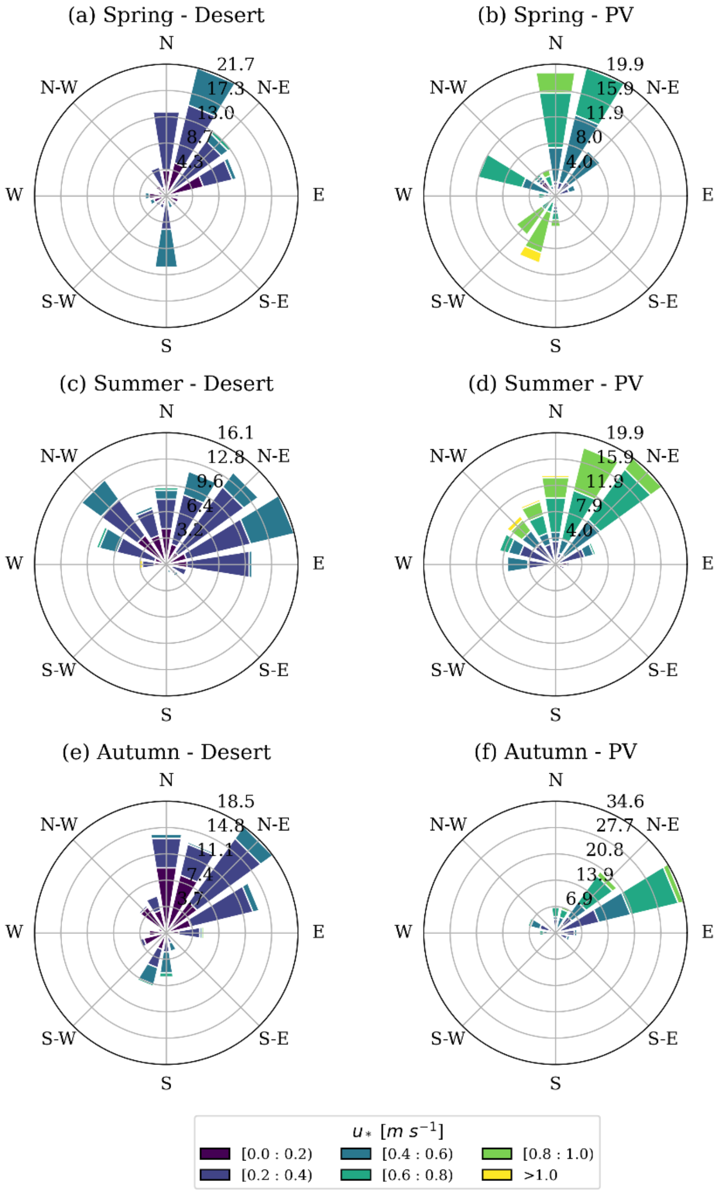

Figure S7: Example of the segmentation of one UAV image

Example of the segmentation of one UAV image, where the bright areas represent: a) complete image with all the elements, b) PV panels only (covering 51% of the rows), c) sun-lit soil and d) shaded soil. This segmentation was performed in every image obtained with the UAV thermal camera, but does not include the infrastructure that is also part of the PV field (Methods S5).

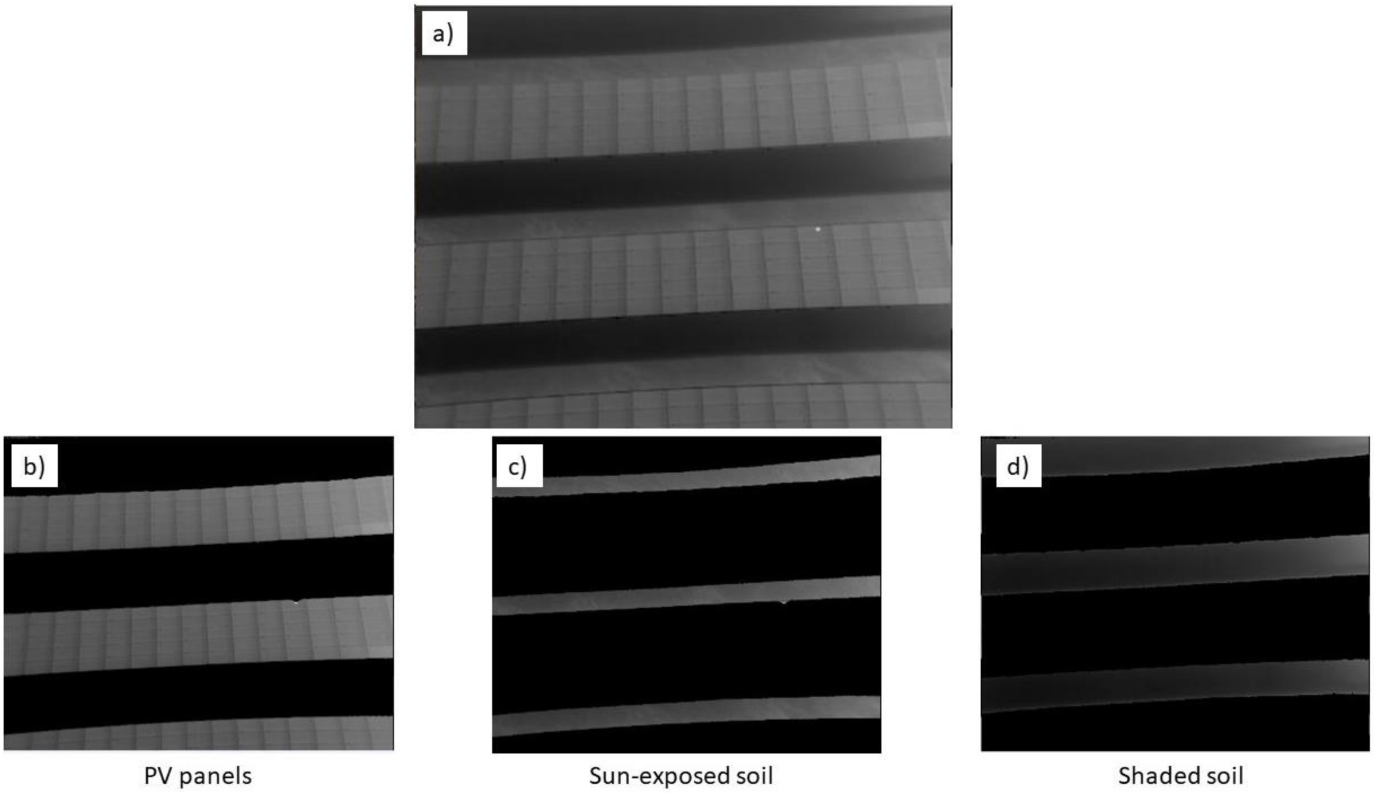

Supplementary Tables

<u>Table S1: Literature review of previous studies on effects of large-scale PV field installations</u>

Summary of the main literature on the geophysical and geochemical effects of PV panels, including the methodology used, the location, the parameters analyzed, and the effect observed, where “>” symbolizes an increase in the effect, and “<” symbolizes a decrease in the effect, and numbers in parenthesis indicate a precise quantification outcome, when present.

Study	Method	Location	Parameter	Effect
Nemet et al., 2009(Nemet, 2009)	Simple equations	Global	C emission suppression	30× albedo change
Millstein and Menon, 2011(Millstein and Menon, 2011)	Fully coupled regional climate models	Desert (USA)	Local surface T_a	> 0.4 °C
Scherba et al., 2011(Scherba *et al.*, 2011)	Model	City (USA)	H<br>Roof temperature	> (up to 405 W m^{-2})<br><
Taha 2013(Taha, 2013)	Meteorological modeling	City (USA)	Local surface T_a	< (up to 0.2 °C)
Masson et al., 2014(Masson *et al.*, 2014)	Town Energy balance scheme	City (France)	Local surface T_a	< (up to 4 °C)
Hu et al., 2016(Hu *et al.*, 2016)	Idealized climate model sensitivity experiments	Desert<br>Global	Local surface T_a<br>Temperature	< (up to 1 °C)<br>Negligible
Salamanca et al., 2016(Salamanca *et al.*, 2016)	Regional climate model	City (USA)	Local surface T_a	< (up to 0.4 °C)
Yang et al., 2017(Yang *et al.*, 2017)	Meteorological and radiation measurements	Desert (China)	Local surface T_a<br>L_{out}	> (up to 1 °C)<br>< (up to 5.7 W m^{-2})
Nguyen et al., 2017(Nguyen *et al.*, 2017)	Atmospheric general circulation model	Australia	Local surface T_a<br>Precipitation	><br>>
Li et al., 2018(Li *et al.*, 2018)	Model with dynamic vegetation	Sahel	Local surface T_a<br>Precipitation	><br>>
Chang et al., 2018(Chang *et al.*, 2018)	Meteorological and radiation measurements	Desert (China)	Local surface T_a<br>L_{out}	><br>< (up to 2.6 W m^{-2})

Broadbent et al., 2019(Broadbent *et al.*, 2019)	Eddy covariance measurements	Desert (USA)	Local surface T_a<br>H	> (up to 1.3 °C)<br>>
Lu et al., 2020(Lu *et al.*, 2020)	Earth System Model	Global	Local surface T_a	> (up to 1.5 °C)
Zhang and Xu 2020(Zhang and Xu, 2020)	Remote Sensing	Global	Local T_s	< (up to 0.53 °C)

Table S2: Mobile system measurements sample size

Season	Site	# measurements half-hours	% half-hours gapfilled
Spring	PV field	191	0.70
	PV background	143	0.25
Autumn	PV field	287	2.79
	PV background	355	0.90
Summer	PV field	287	1.05
	PV background	275	2.55

Table S3: Aerodynamic resistance to heat transfer for Autumn

	PV		Afforestation
Land-use	PV field	Background	Forest
R_n (W m^{-2})	361 (98)	260 (68)	439 (135)
H (W m^{-2})	251 (48)	144 (31)	353 (118)
LE (W m^{-2})	21 (8)	27 (16)	37 (36)
L_{out} (W m^{-2})	515 (7)	526 (10)	463 (20)
T_s (°C)	39.8 (1.2)	40.1 (1.5)	30.3 (3.6)
T_a (°C)	29.0 (2.1)	31.7 (2.4)	25.6 (3.4)
ΔT_{s-a} (°C)	10.8 (1.9)	8.4 (2.2)	4.4 (1.3)
r_H (s m^{-1})	50.0 (11.0)	66.0 (16.0)	14.0 (5.0)

Table S4: Aerodynamic resistance to heat transfer for Spring

	PV		Afforestation	
Land-use	PV field	Background	Forest	Background
R_n (W m^{-2})	500 (104)	344 (53)	470 (172)	434 (109)
H (W m^{-2})	178 (61)	173 (37)	246 (114)	134 (59)
LE (W m^{-2})	12 (17)	10 (8)	129 (56)	122 (58)
L_{out} (W m^{-2})	507 (29)	519 (13)	414 (28)	452 (31)
T_s (°C)	38.2 (4.5)	39.9 (2.0)	21.6 (5.8)	26.4 (5.4)
T_a (°C)	28.3 (4.7)	30.9 (2.3)	18.0 (4.8)	18.2 (4.2)
ΔT_{s-a} (°C)	9.9 (2.5)	9.1 (1.3)	3.9 (1.8)	8.2 (3.0)
r_H (s m^{-1})	67.0 (22.0)	62.0 (24.0)	20.0 (7.0)	77.0 (41.0)

References

Bradski, G. (2000) 'The OpenCV Library', *Dr. Dobb's Journal of Software Tools*.

Broadbent, A. M. *et al.* (2019) 'The observed effects of utility-scale photovoltaics on near-surface air temperature and energy balance', *Journal of Applied Meteorology and Climatology*, 58(5), pp. 989–1006. doi: 10.1175/JAMC-D-18-0271.1.

Burg, B. R. *et al.* (2015) 'Placement and efficiency effects on radiative forcing of solar installations', *AIP Conference Proceedings*, 1679(September). doi: 10.1063/1.4931546.

Chang, R. *et al.* (2018) 'Observed surface radiation and temperature impacts from the large-scale deployment of photovoltaics in the barren area of Gonghe, China', *Renewable Energy*. Elsevier Ltd, 118, pp. 131–137. doi: 10.1016/j.renene.2017.11.007.

Falk, T. *et al.* (2019) 'U-Net: deep learning for cell counting, detection, and morphometry', *Nature Methods*, 16(1), pp. 67–70. doi: 10.1038/s41592-018-0261-2.

GPPD (2018) *Global Power Plant Database - Datasets - Data*. Resource Watch and Google Earth Engine. Available at: https://datasets.wri.org/dataset/globalpowerplantdatabase.

Hu, A. *et al.* (2016) 'Impact of solar panels on global climate', *Nature Climate Change*, 6(3), pp. 290–294. doi: 10.1038/nclimate2843.

Incropera, F. P. *et al.* (1996) *Fundamentals of Heat and Mass Transfer*. Sixth. Hoboken, New Jersey: John Wiley & Sons, Ltd. doi: 10.1016/j.applthermaleng.2011.03.022.

Kawajiri, K., Oozeki, T. and Genchi, Y. (2011) 'Effect of temperature on PV potential in the world', *Environmental Science and Technology*, 45(20), pp. 9030–9035. doi: 10.1021/es200635x.

Li, Y. *et al.* (2018) 'Climate model shows large-scale wind and solar farms in the Sahara increase rain and vegetation', *Science*, 361(6406), pp. 1019–1022. doi: 10.1126/science.aar5629.

Lu, Z. *et al.* (2020) 'Impacts of Large-Scale Sahara Solar Farms on Global Climate and Vegetation Cover', *Geophysical Research Letters*, 48(2), pp. 1–10. doi: 10.1029/2020GL090789.

Luyssaert, S. *et al.* (2007) 'CO 2 balance of boreal, temperate, and tropical forests derived from a global database', *Global Change Biology*, 13(12), pp. 2509–2537. doi: 10.1111/j.1365-

2486.2007.01439.x.

Maes, W. H. *et al.* (2019) 'Potential evaporation at eddy-covariance sites across the globe', *Hydrology and Earth System Sciences*, 23(2), pp. 925–948. doi: 10.5194/hess-23-925-2019.

Masson, V. *et al.* (2014) 'Solar panels reduce both global warming and urban heat island', *Frontiers in Environmental Science*, 2(June), pp. 1–10. doi: 10.3389/fenvs.2014.00014.

Millstein, D. and Menon, S. (2011) 'Regional climate consequences of large-scale cool roof and photovoltaic array deployment', *Environmental Research Letters*, 6(3). doi: 10.1088/1748-9326/6/3/034001.

Nemet, G. F. (2009) 'Net radiative forcing from widespread deployment of photovoltaics', *Environmental Science and Technology*, 43(6), pp. 2173–2178. doi: 10.1021/es801747c.

Nguyen, K. C. *et al.* (2017) 'Potential impacts of solar arrays on regional climate and on array efficiency', *International Journal of Climatology*, 37(11), pp. 4053–4064. doi: 10.1002/joc.4995.

Salamanca, F. *et al.* (2016) 'Citywide Impacts of Cool Roof and Rooftop Solar Photovoltaic Deployment on Near-Surface Air Temperature and Cooling Energy Demand', *Boundary-Layer Meteorology*. Springer Netherlands, 161(1), pp. 203–221. doi: 10.1007/s10546-016-0160-y.

Scherba, A. *et al.* (2011) 'Modeling impacts of roof reflectivity, integrated photovoltaic panels and green roof systems on sensible heat flux into the urban environment', *Building and Environment*. Elsevier Ltd, 46(12), pp. 2542–2551. doi: 10.1016/j.buildenv.2011.06.012.

Sekertekin, A. and Bonafoni, S. (2020) 'Land surface temperature retrieval from Landsat 5, 7, and 8 over rural areas: Assessment of different retrieval algorithms and emissivity models and toolbox implementation', *Remote Sensing*, 12(2). doi: 10.3390/rs12020294.

SPA (2021) *Science Panel for the Amazon*.

Taha, H. (2013) 'The potential for air-temperature impact from large-scale deployment of solar photovoltaic arrays in urban areas', *Solar Energy*. Elsevier Ltd, 91, pp. 358–367. doi: 10.1016/j.solener.2012.09.014.

Thakur, G. *et al.* (2022) 'Downwelling longwave radiation and sensible heat flux observations are critical for surface temperature and emissivity estimation from flux tower data', *Scientific Reports*.

Nature Publishing Group UK, pp. 1–14. doi: 10.1038/s41598-022-12304-3.

Trabucco, A. and Zomer, R. J. (2018) 'Global Aridity Index and Potential Evapotranspiration (ET0) Climate Database v2', *CGIAR Consortium for Spatial Information (CGIAR-CSI)*, (November), p. 10. doi: 10.6084.

Tsvetsinskaya, E. A. *et al.* (2006) 'Spatial and temporal variability in Moderate Resolution Imaging Spectroradiometer-derived surface albedo over global arid regions', *Journal of Geophysical Research Atmospheres*, 111(20), pp. 1–10. doi: 10.1029/2005JD006772.

Vishnevetsky, I. *et al.* (2019) 'Method for accurate measurement of infrared emissivity for opaque low-reflectance materials', *Applied Optics*, 58(17), pp. 4599–4609. doi: 10.1364/AO.58.004599.

Yang, L. *et al.* (2017) 'Study on the local climatic effects of large photovoltaic solar farms in desert areas', *Solar Energy*. Elsevier Ltd, 144, pp. 244–253. doi: 10.1016/j.solener.2017.01.015.

Zhang, X. and Xu, M. (2020) 'Assessing the effects of photovoltaic powerplants on surface temperature using remote sensing techniques', *Remote Sensing*, 12(11), pp. 8–14. doi: 10.3390/rs12111825.
